# Using Perturbed Underdamped Langevin Dynamics to Efficiently Sample from Probability Distributions

**DOI:** 10.1007/s10955-017-1906-8

**Published:** 2017-11-02

**Authors:** A. B. Duncan, N. Nüsken, G. A. Pavliotis

**Affiliations:** 1 School of Mathematical and Physical Sciences, University of Sussex, Falmer, Brighton, BN1 9RH UK; 2 Department of Mathematics, Imperial College London, South Kensington Campus, London, SW7 2AZ England, UK

## Abstract

In this paper we introduce and analyse Langevin samplers that consist of perturbations of the standard underdamped Langevin dynamics. The perturbed dynamics is such that its invariant measure is the same as that of the unperturbed dynamics. We show that appropriate choices of the perturbations can lead to samplers that have improved properties, at least in terms of reducing the asymptotic variance. We present a detailed analysis of the new Langevin sampler for Gaussian target distributions. Our theoretical results are supported by numerical experiments with non-Gaussian target measures.

## Introduction and Motivation

Sampling from probability measures in high-dimensional spaces is a problem that appears frequently in applications, e.g. in computational statistical mechanics and in Bayesian statistics. In particular, we are faced with the problem of computing expectations with respect to a probability measure $\pi$ on $\mathbb {R}^{d}$, i.e. we wish to evaluate integrals of the form:

1 $$\begin{aligned} \pi (f):=\int _{\mathbb {R}^{d}}f(x)\pi (\mathrm {d}x). \end{aligned}$$

As is typical in many applications, particularly in molecular dynamics and Bayesian inference, the density (for convenience denoted by the same symbol $\pi$) is known only up to a normalization constant; furthermore, the dimension of the underlying space is quite often large enough to render deterministic quadrature schemes computationally infeasible.

A standard approach to approximating such integrals is Markov Chain Monte Carlo (MCMC) techniques [19, 32, 52], where a Markov process $(X_{t})_{t\ge 0}$ is constructed which is ergodic with respect to the probability measure $\pi$. Then, defining the long-time average

2 $$\begin{aligned} \pi _{T}(f):=\frac{1}{T}\int _{0}^{T}f(X_{s})\mathrm {d}s \end{aligned}$$

for $f\in L^{1}(\pi )$, the ergodic theorem guarantees almost sure convergence of the long-time average $\pi _{T}(f)$ to $\pi (f)$.

There are infinitely many Markov, and, for the purposes of this paper diffusion, processes that can be constructed in such a way that they are ergodic with respect to the target distribution. A natural question is then how to choose the ergodic diffusion process $(X_{t})_{t\ge 0}$. Naturally the choice should be dictated by the requirement that the computational cost of (approximately) calculating (1) is minimized. A standard example is given by the *overdamped Langevin dynamics* defined to be the unique (strong) solution $(X_{t})_{t\ge 0}$ of the following stochastic differential equation (SDE):

3 $$\begin{aligned} \mathrm {d}X_{t}=-\nabla V(X_{t})\mathrm {d}t+\sqrt{2}\mathrm {d}W_{t}, \end{aligned}$$

where $V=-\log \pi$ is the potential associated with the smooth positive density $\pi$. Under appropriate assumptions on *V*, i.e. on the measure $\pi (\mathrm {d}x)$, the process $(X_{t})_{t\ge 0}$ is ergodic and in fact reversible with respect to the target distribution.

Another well-known example is the *underdamped Langevin dynamics* given by $(X_t)_{t\ge 0} = (q_t, p_t)_{t\ge 0}$ defined on the extended space (phase space) $\mathbb {R}^{d}\times \mathbb {\mathbb {R}}^{d}$ by the following pair of coupled SDEs:

4 $$\begin{aligned} \mathrm {d}q_{t} =M^{-1}p_{t}\mathrm {d}t, \quad \mathrm {d}p_{t} =-\nabla V(q_{t})\mathrm {d}t-\varGamma M^{-1}p_{t}\mathrm {d}t+\sqrt{2\varGamma }\mathrm {d}W_{t}, \end{aligned}$$

where the mass and friction tensors *M* and $\varGamma$ are assumed to be symmetric positive definite matrices. It is well-known [36, 46] that $(q_{t},p_{t})_{t\ge 0}$ is ergodic with respect to the measure $\widehat{\pi }:=\pi \otimes \mathcal {N}(0,M)$, having density with respect to the Lebesgue measure on $\mathbb {R}^{2d}$ given by

5 $$\begin{aligned} \widehat{\pi }(q,p)=\frac{1}{\widehat{Z}}\exp \left( -V(q)-\frac{1}{2}p\cdot M^{-1}p\right) , \end{aligned}$$

where $\widehat{Z}$ is a normalization constant. Note that $\widehat{\pi }$ has marginal $\pi$ with respect to *p* and thus for functions $f\in L^{1}(\pi )$, we have that $\frac{1}{t}\int _0^t f(q_{t})\,\mathrm {d}t\rightarrow \pi (f)$ almost surely. Notice also that the dynamics restricted to the *q*-variables is no longer Markovian. The *p*-variables can thus be interpreted as giving some instantaneous memory to the system, facilitating efficient exploration of the state space. Higher order Markovian models, based on a finite dimensional (Markovian) approximation of the generalized Langevin equation can also be used [12].

As there is a lot of freedom in choosing the dynamics in (2), see the discussion in Sect. 2, it is desirable to choose the diffusion process $(X_t)_{t\ge 0}$ in such a way that $\pi _T(f)$ can provide a good estimation of $\pi (f)$. The performance of the estimator (2) can be quantified in various manners. The ultimate goal, of course, is to choose the dynamics as well as the numerical discretization in such a way that the computational cost of the longtime-average estimator is minimized, for a given tolerance. The minimization of the computational cost consists of three steps: bias correction, variance reduction and choice of an appropriate discretization scheme. For the latter step see Sect. 5 and [14, Sect. 6].

Under appropriate conditions on the potential *V* it can be shown that both (3) and (4) converge to equilibrium exponentially fast, e.g. in relative entropy. One performance objective would then be to choose the process $(X_t)_{t\ge 0}$ so that this rate of convergence is maximised. Conditions on the potential *V* which guarantee exponential convergence to equilibrium, both in $L^{2}(\pi )$ and in relative entropy can be found in [7, 39, 54]. In the case when the target measure $\pi$ is Gaussian, both the overdamped  (3) and the underdamped (4) dynamics become generalized Ornstein–Uhlenbeck processes. For such processes the entire spectrum of the generator—or, equivalently, the Fokker–Planck operator—can be computed analytically and, in particular, an explicit formula for the $L^2$-spectral gap can be obtained [38, 43, 44]. A detailed analysis of the convergence to equilibrium in relative entropy for stochastic differential equations with linear drift, i.e. generalized Ornstein–Uhlenbeck processes, has been carried out in [1, 2].

In addition to speeding up convergence to equilibrium, i.e. reducing the bias of the estimator (2), one is naturally also interested in reducing the asymptotic variance. Under appropriate conditions on the target measure $\pi$ and the observable *f*, the estimator $\pi _T(f)$ satisfies a central limit theorem (CLT) [31], that is,

$$\begin{aligned} \frac{1}{\sqrt{T}}\left( \pi _T(f) - \pi (f)\right) \xrightarrow [T\rightarrow \infty ]{d} \mathcal {N}\left( 0, 2\,\sigma ^2_f\right) , \end{aligned}$$

where $\sigma ^2_f < \infty$ is the *asymptotic variance* of the estimator $\pi _T(f)$. The asymptotic variance characterises the magnitude of fluctuations of $\pi _T(f)$ around $\pi (f)$. Consequently, another natural objective is to choose the process $(X_t)_{t\ge 0}$ such that $\sigma ^2_f$ is as small as possible. It is well known that the asymptotic variance can be expressed in terms of the solution to an appropriate Poisson equation for the generator of the dynamics [31]

6 $$\begin{aligned} - \mathcal L\phi = f - \pi (f), \quad \sigma ^2_f = \int _{\mathbb {R}^d} \phi (- \mathcal L\phi ) \, \pi (\mathrm {d}x). \end{aligned}$$

Techniques from the theory of partial differential equations can then be used in order to study the problem of minimizing the asymptotic variance. This is the approach that was taken in [14], see also [23], and it will also be used in this paper.

Other measures of performance have also been considered. For example, in [50, 51], performance of the estimator is quantified in terms of the rate functional of the ensemble measure $\frac{1}{t}\int _0^t \delta _{X(t)}(dx)$. See also [28] for a study of the nonasymptotic behaviour of MCMC techniques, including the case of overdamped Langevin dynamics.

Similar analyses have been carried out for various modifications of (3). Of particular interest to us are the *Riemannian manifold MCMC* [18] (see the discussion in Sect. 2) and the *nonreversible Langevin samplers* [20, 21]. As a particular example of the general framework that was introduced in [18], we mention the preconditioned overdamped Langevin dynamics $\mathrm {d}X_t = -P \nabla V(X_t)\,\mathrm {d}t + \sqrt{2P}\,\mathrm {d}W_t,$ that was presented in [4]. There, the long-time behaviour of as well as the asymptotic variance of the corresponding estimator $\pi _T(f)$ are studied and applied to equilibrium sampling in molecular dynamics. A variant of the standard underdamped Langevin dynamics that can be thought of as a form of preconditioning and that has been used by practitioners is the *mass-tensor molecular dynamics* [6].

The nonreversible overdamped Langevin dynamics

7 $$\begin{aligned} \mathrm {d}X_t = -\left( \nabla V(X_t) - \gamma (X_t)\right) \,\mathrm {d}t + \sqrt{2}\,\mathrm {d}W_t, \end{aligned}$$

where the vector field $\gamma$ satisfies $\nabla \cdot (\pi \gamma ) = 0$ is ergodic (but not reversible) with respect to the target measure $\pi$ for all choices of the divergence-free vector field $\gamma$. The asymptotic behaviour of this process was considered for Gaussian diffusions in [20], where the rate of convergence of the covariance to equilibrium was quantified in terms of the choice of $\gamma$. This work was extended to the case of non-Gaussian target densities, and consequently for nonlinear SDEs of the form (7) in [21]. The problem of constructing the optimal nonreversible perturbation, in terms of the $L^2(\pi )$ spectral gap for Gaussian target densities was studied in  [34] see also [55]. Optimal nonreversible perturbations with respect to miniziming the asymptotic variance were studied in [14, 23]. In all these works it was shown that, in theory [i.e. without taking into account the computational cost of the discretization of the dynamics (7)], the nonreversible Langevin sampler (7) is never worse that its reversible counterpart (3), both in terms of converging faster to the target distribution as well as in terms of having a lower asymptotic variance. It should be emphasized that the two optimality criteria, maximizing the spectral gap and minimizing the asymptotic variance, lead to different choices for the nonreversible drift $\gamma (x)$.

The goal of this paper is to extend the analysis presented in [14, 34] by introducing the following modification of the standard underdamped Langevin dynamics:

8 $$\begin{aligned} \mathrm {d}q_{t}= & {} M^{-1}p_{t}\mathrm {d}t-\mu J_{1}\nabla V(q_{t})\mathrm {d}t, \nonumber \\ \mathrm {d}p_{t}= & {} -\nabla V(q_{t})\mathrm {d}t-\nu J_{2}M^{-1}p_{t}\mathrm {d}t-\varGamma M^{-1}p_{t}\mathrm {d}t+\sqrt{2\varGamma }\mathrm {d}W_{t}, \end{aligned}$$

where $M,\varGamma \in \mathbb {R}^{d\times d}$ are constant strictly positive definite matrices, $\mu$ and $\nu$ are scalar constants and $J_1, J_2 \in \mathbb {R}^{d\times d}$ are constant skew-symmetric matrices. As demonstrated in Sect. 2, the process defined by (8) will be ergodic with respect to the Gibbs measure $\widehat{\pi }$ defined in (5).

Our objective is to investigate the use of these dynamics for computing ergodic averages of the form (2). To this end, we study the long time behaviour of (8) and, using hypocoercivity techniques, prove that the process converges exponentially fast to equilibrium. This perturbed underdamped Langevin process introduces a number of parameters in addition to the mass and friction tensors which must be tuned to ensure that the process is an efficient sampler. For Gaussian target densities, we derive estimates for the spectral gap and the asymptotic variance, valid in certain parameter regimes. Moreover, for certain classes of observables, we are able to identify the choices of parameters which lead to the optimal performance in terms of asymptotic variance. While these results are valid for Gaussian target densities, we advocate these particular parameter choices also for more complex target densities. To demonstrate their efficacy, we perform a number of numerical experiments on more complex, multimodal distributions. In particular, we use the Langevin sampler (8) in order to study the problem of diffusion bridge sampling.

The rest of the paper is organized as follows. In Sect. 2 we present some background material on Langevin dynamics, we construct general classes of Langevin samplers and we introduce criteria for assessing the performance of the samplers. In Sect. 3 we study qualitative properties of the perturbed underdamped Langevin dynamics (8) including exponentially fast convergence to equilibrium and the overdamped limit. In Sect. 4 we study in detail the performance of the Langevin sampler (8) for the case of Gaussian target distributions. In Sect. 5 we introduce a numerical scheme for simulating the perturbed dynamics (8) and we present numerical experiments on the implementation of the proposed samplers for the problem of diffusion bridge sampling. Section 6 is reserved for conclusions and suggestions for further work.

## Construction of General Langevin Samplers

### Background and Preliminaries

In this section we consider estimators of the form (2) where $(X_t)_{t\ge 0}$ is a diffusion process given by the solution of the following Itô SDE:

9 $$\begin{aligned} \mathrm {d}X_t = a(X_t)\,\mathrm {d}t + \sqrt{2}b(X_t)\,\mathrm {d}W_t, \end{aligned}$$

with drift coefficient $a: {{\mathrm{\mathbb {R}}}}^d \rightarrow {{\mathrm{\mathbb {R}}}}^d$ and diffusion coefficient $b:{{\mathrm{\mathbb {R}}}}^d \rightarrow {{\mathrm{\mathbb {R}}}}^{d\times m}$ both having smooth components, and where $(W_t)_{t\ge 0}$ is a standard ${{\mathrm{\mathbb {R}}}}^m$–valued Brownian motion. Associated with (9) is the infinitesimal generator ${{\mathrm{\mathcal {L}}}}$ formally given by

10 $$\begin{aligned} {{\mathrm{\mathcal {L}}}}f = a\cdot \nabla f + \varSigma : \nabla \nabla f, \quad f \in C^2_c({{\mathrm{\mathbb {R}}}}^d) \end{aligned}$$

where $\varSigma = bb^\top$, $\nabla \nabla f$ denotes the Hessian of the function *f* and $\, : \,$ denotes the Frobenius inner product. In general, $\varSigma$ is nonnegative definite, and could possibly be degenerate. In particular, the infinitesimal generator (10) need not be uniformly elliptic. To ensure that the corresponding semigroup exhibits sufficient smoothing behaviour, we shall require that the process (9) is hypoelliptic in the sense of Hörmander. If this condition holds, then irreducibility of the process $(X_t)_{t\ge 0}$ will be an immediate consequence of the existence of a strictly positive invariant distribution $\pi (x)\mathrm {d}x$, see [30].

Suppose that $(X_t)_{t\ge 0}$ is nonexplosive. It follows from the hypoellipticity assumption that the process $(X_t)_{t\ge 0}$ possesses a smooth transition density *p*(*t*, *x*, *y*) which is defined for all $t \ge 0$ and $x, y \in {{\mathrm{\mathbb {R}}}}^d$, [5, Theorem VII.5.6]. The associated strongly continuous Markov semigroup $(P_t)_{t\ge 0}$ is defined by $P_t f(x) = \int _{{{\mathrm{\mathbb {R}}}}^d} p(t, x, y)f(y)\,\mathrm {d}y.$ Suppose that $(P_t)_{t\ge 0}$ is invariant with respect to the target measure $\pi$, i.e. $\int _{\mathbb {R}^d} P_t f(x)\pi (\mathrm {d}x) = \int _{\mathbb {R}^d} f(x)\pi (\mathrm {d}x)$ for $t\ge 0$ and all bounded continuous functions *f*. Then $(P_t)_{t\ge 0}$ can be extended to a positivity preserving contraction semigroup on $L^2(\pi )$ which is strongly continuous. Moreover, the infinitesimal generator corresponding to $(P_t)_{t\ge 0}$ is given by an extension of $({{\mathrm{\mathcal {L}}}}, C^{2}_c({{\mathrm{\mathbb {R}}}}^d))$, also denoted by ${{\mathrm{\mathcal {L}}}}$.

Due to hypoellipticity and invariance with respect to $(P_t)_{t \ge 0}$, the probability measure $\pi$ on $\mathbb {R}^d$ has a smooth density with respect to the Lebesgue measure. If this density is strictly positive, it follows that $\pi$ is necessarily the unique invariant distribution. Slightly abusing the notation, we will denote both the measure and its density by $\pi$. Furthermore, we will denote by $L^2(\pi )$ be the Hilbert space of $\pi$-square integrable functions equipped with inner product $\left\langle \cdot , \cdot \right\rangle _{L^2(\pi )}$ and norm $\left||{\cdot }\right||_{L^2(\pi )}$.

### A General Characterisation of Ergodic Diffusions

A natural question is what conditions on the coefficients *a* and *b* of (9) are required to ensure that $(X_t)_{t\ge 0}$ is invariant with respect to the distribution $\pi (x)\,\mathrm {d}x$. The following result provides a necessary and sufficient condition for a diffusion process to be invariant with respect to a given target distribution.

#### Theorem 1

Consider a diffusion process $(X_t)_{t\ge 0}$ on $\mathbb {R}^{d}$ defined by the unique, non-explosive solution to the Itô SDE (9) with drift $a \in C^1(\mathbb {R}^{d}; \mathbb {R}^d)$ and diffusion coefficient $b\in C^1(\mathbb {R}^d; \mathbb {R}^{d\times m})$. Then $(X_t)_{t\ge 0}$ is invariant with respect to ${\pi }$ if and only if

11 $$\begin{aligned} a = \varSigma \nabla \log \pi + \nabla \cdot \varSigma + \gamma , \end{aligned}$$

where $\varSigma = bb^\top$ and $\gamma : \mathbb {R}^D\rightarrow \mathbb {R}^D$ is a continuously differentiable vector field satisfying

12 $$\begin{aligned} \nabla \cdot \left( \pi \gamma \right) = 0. \end{aligned}$$

If additionally $\gamma \in L^1({\pi })$, then there exists a skew-symmetric matrix function $C:\mathbb {R}^d \rightarrow \mathbb {R}^{d\times d}$ such that $\gamma = \frac{1}{{\pi }} \nabla \cdot \left( {\pi } C \right) .$ In this case the infinitesimal generator can be written as an $L^2(\pi )$-extension of

$$\begin{aligned} \mathcal {L}f = \frac{1}{{\pi }}\nabla \cdot \left( (\varSigma + C){\pi }\nabla f\right) ,\quad f\in C^2_c(\mathbb {R}^d). \end{aligned}$$

The proof of the first part of this result can be found in [46, Chap. 4]; similar versions of this characterisation can be found in [54] and [21]. For the existence of the skew-symmetric matrix *C* see, e.g., [16, Sec.4, Prop. 1]. See also [37].

#### Remark 1

If (11) holds and $\mathcal {L}$ is hypoelliptic it follows immediately that $(X_t)_{t\ge 0}$ is ergodic with unique invariant distribution $\pi (x)\,\mathrm {d}x$ (see [30]).

More generally, we can consider Itô diffusions in an extended phase space:

13 $$\begin{aligned} \mathrm {d} Z_{t} = b(Z_t) \, \mathrm {d}t + \sqrt{2}\sigma (Z_{t}) \, \mathrm {d}W_{t}, \end{aligned}$$

where $(W_{t})_{t\ge 0}$ is a standard Brownian motion in ${{\mathrm{\mathbb {R}}}}^{N}$, $N \ge d$. This is a Markov process with generator

14 $$\begin{aligned} \mathcal {L} = b(z) \cdot \nabla _z + \varSigma (z) : \nabla _z \nabla _z, \end{aligned}$$

where $\varSigma (z) = \big ( \sigma \sigma ^{T} \big )(z)$. We will consider dynamics $(Z_t)_{t\ge 0}$ that is ergodic with respect to $\pi _z(z) \, \mathrm {d}z$ such that $\int _{{{\mathrm{\mathbb {R}}}}^{m}} \pi _z (x, \, y) \, \mathrm {d}y = \pi (x),$ where $z = (x, \, y), \; x \in {{\mathrm{\mathbb {R}}}}^d, \, y \in {{\mathrm{\mathbb {R}}}}^m, \; d+m = N$.

There are various well-known choices of dynamics which are invariant (and indeed ergodic) with respect to the target distribution $\pi (x)\mathrm {d}x$.

1. Choosing $b = I$ and $\gamma = 0$ we immediately recover the overdamped Langevin dynamics (3).
2. Choosing $b = I$, and $\gamma \ne 0$ such that (12) holds gives rise to the nonreversible overdamped equation defined by (7). As it satisfies the conditions of Theorem 1, it is ergodic with respect to $\pi$. In particular choosing $\gamma (x) = J\nabla V(x)$ for a constant skew-symmetric matrix *J* we obtain
  15 $$\begin{aligned} \mathrm {d}X_t = -(I + J)\nabla V(X_t)\,\mathrm {d}t + \sqrt{2}\,\mathrm {d}W_t, \end{aligned}$$
  which has been studied in previous works.
3. Given a target density $\pi > 0$ on ${{\mathrm{\mathbb {R}}}}^d$, if we consider the augmented target density $\widehat{\pi }$ on ${{\mathrm{\mathbb {R}}}}^{2d}$ given in (5), then choosing
  16 $$\begin{aligned} \gamma ((q,p)) = \left( \begin{array}{c} M^{-1}p \\ -\nabla V(q)\end{array}\right) \quad \text{ and }\quad b = \left( \begin{array}{c}\varvec{0} \\ \sqrt{\varGamma }\end{array}\right) \in \mathbb {R}^{2d \times d}, \end{aligned}$$
  where *M* and $\varGamma$ are positive definite symmetric matrices, the conditions of Theorem 1 are satisfied for the target density $\widehat{\pi }$. The resulting dynamics $(q_t, p_t)_{t\ge 0}$ is determined by the underdamped Langevin equation (4). It is straightforward to verify that the generator is hypoelliptic, [35, Sec 2.2.3.1], and thus $(q_t, p_t)_{t\ge 0}$ is ergodic.
4. More generally, consider the augmented target density $\widehat{\pi }$ on $\mathbb {R}^{2d}$ as above, and choose
  17 $$\begin{aligned} \gamma ((q,p)) = \left( \begin{array}{c} M^{-1}p - \mu J_1\nabla V(q) \\ -\nabla V(q) - \nu J_2 M^{-1}p\end{array}\right) \quad \text{ and }\quad b = \left( \begin{array}{c}\varvec{0} \\ \sqrt{\varGamma }\end{array}\right) \in \mathbb {R}^{2d \times d}, \end{aligned}$$
  where $\mu$ and $\nu$ are scalar constants and $J_1, J_2 \in \mathbb {R}^{d\times d}$ are constant skew-symmetric matrices. With this choice we recover the perturbed Langevin dynamics (8). It is straightforward to check that (17) satisfies the invariance condition (12), and thus Theorem 1 guarantees that (8) is invariant with respect to $\widehat{\pi }$.
5. In a similar fashion, one can introduce an augmented target density on $\mathbb {R}^{(m+2)d}$, with
  $$\begin{aligned} \widehat{\widehat{\pi }}(q, p, u_1,\ldots , u_m) \propto e^{-\left( \frac{|p|^2}{2} + \frac{\vert u_1 \vert ^2 + \ldots + \vert u_m \vert ^2}{2}+V(q)\right) }, \end{aligned}$$
  where $p, q, u_i \in \mathbb {R}^d$, for $i=1,\ldots , m$. Clearly $\int _{\mathbb {R}^{d}\times \mathbb {R}^{md}} \widehat{\widehat{\pi }}(q, p, u_1,\ldots ,u_m)\,\mathrm {d}p\,\mathrm {d}u_1\,\ldots \mathrm {d}u_m = \pi (q)$. We now define $\gamma :\mathbb {R}^{(m+2)d}\rightarrow \mathbb {R}^{(m+2)d}$ by
  $$\begin{aligned} \gamma (q,p, u_1,\ldots ,u_m) = \left( p \quad -\nabla _q V(q) + \sum _{j=1}^{m} \lambda _j u_j \quad -\lambda _1 p \quad \cdots \quad -\lambda _m p \right) ^{T} \end{aligned}$$
  and $$b: \mathbb {R}^{(m+2)d}\rightarrow \mathbb {R}^{(m+2)d\times (m+2)d}$$ by
  $$\begin{aligned} b(q,p,u_1,\ldots , u_m) = \left( \begin{array}{c@{\quad }c@{\quad }c@{\quad }c@{\quad }c@{\quad }c}\varvec{0} &{} \varvec{0} &{} \varvec{0} &{} \varvec{0} &{} \ldots &{} \varvec{0}\\ \varvec{0} &{} \varvec{0} &{} \varvec{0} &{} \varvec{0}&{} \ldots &{} \varvec{0} \\ \varvec{0} &{} \varvec{0} &{} \sqrt{\alpha _1}I_{d\times d} &{} \varvec{0} &{} \ldots &{} \varvec{0} \\ \varvec{0} &{} \varvec{0} &{} \varvec{0} &{} \sqrt{\alpha _2}I_{d\times d} &{} \ldots &{} \varvec{0} \\ \vdots &{} \vdots &{} \vdots &{} \vdots &{} \ddots &{} \vdots \\ \varvec{0} &{} \varvec{0} &{} \varvec{0} &{}\varvec{0} &{} \ldots &{} \sqrt{\alpha _m}I_{d\times d}\end{array}\right) , \end{aligned}$$
  where $\lambda _i \in \mathbb {R}$ and $\alpha _i > 0$, for $i=1,\ldots , m$. The resulting process (9) is given by
  18 $$\begin{aligned} \begin{aligned} \mathrm {d}q_t&= p_t \,\mathrm {d}t, \quad \mathrm {d}p_t = -\nabla _q V(q_t)\,\mathrm {d}t + \sum _{j=1}^{d}\lambda _j u^{j}(t)\,\mathrm {d}t, \\ \mathrm {d}u^{1}_t&= -\lambda _1 p_t\,\mathrm {d}t -\alpha _1 u^{1}_t\,\mathrm {d}t + \sqrt{2\alpha _1 }\,\mathrm {d}W^{1}_t,\\ \vdots&\\ \mathrm {d}u^{m}_t&= -\lambda _m p_t\,\mathrm {d}t -\alpha _m u^{m}_t\,\mathrm {d}t + \sqrt{2\alpha _m }\,\mathrm {d}W^{m}_t, \end{aligned} \end{aligned}$$
  where $(W^j_t)_{t\ge 0,j=1,\ldots ,m}$ are independent $\mathbb {R}^d$–valued Brownian motions. This process is ergodic with unique invariant distribution $\widehat{\widehat{\pi }}$, and under appropriate conditions on *V*, converges exponentially fast to equilibrium in relative entropy [42]. Equation (18) is a Markovian representation of a generalised Langevin equation of the form
  $$\begin{aligned} \mathrm {d}q_t = p_t \,\mathrm {d}t, \quad \mathrm {d}p_t = -\nabla _{q}V(q_t) \,\mathrm {d}t - \int _0^t F(t-s)p_s\,\mathrm {d}s + N(t), \end{aligned}$$
  where *N*(*t*) is a mean-zero stationary Gaussian process with autocorrelation function *F*(*t*), i.e. $\mathbb {E}\left[ N(t) \otimes N(s) \right] = F(t-s)I_{d\times d}$ and $F(t) = \sum _{i=1}^{m} \lambda _i^2 e^{-\alpha _i|t|}.$
6. Let $\widetilde{\pi }(z) \propto \exp (-\Phi (z))$ be a positive density on $\mathbb {R}^N$ where $N > d$ such that $\pi (x) = \int _{\mathbb {R}^{N-d}}\widetilde{\pi }(x,z)\,\mathrm {d}z,$ where $(x, y)\in \mathbb {R}^d\times \mathbb {R}^{N-d}$. Then choosing $b = I_{D\times D}$ and $\gamma = 0$ we obtain the dynamics
  $$\begin{aligned} \mathrm {d}X_t = -\nabla _x \Phi (X_t, Y_t)\,\mathrm {d}t + \sqrt{2}\,\mathrm {d}W^{1}_t, \quad \mathrm {d}Y_t = -\nabla _y \Phi (X_t, Y_t)\,\mathrm {d}t + \sqrt{2}\,\mathrm {d}W^{2}_t, \end{aligned}$$
  then $(X_t, Y_t)_{t\ge 0}$ is immediately ergodic with respect to $\widetilde{\pi }$.

### Comparison Criteria

For a fixed observable *f*, a natural measure of accuracy of the estimator $\pi _T(f) = t^{-1}\int _0^{t}f(X_s)\,\mathrm {d}s$ is the *mean square error* (MSE) defined by

19 $$\begin{aligned} MSE(f, T) := \mathbb {E}_{x}\left| {\pi _T(f) - \pi (f)}\right| ^2, \end{aligned}$$

where $\mathbb {E}_{x}$ denotes the expectation conditioned on the process $(X_t)_{t\ge 0}$ starting at *x*. It is instructive to introduce the decomposition $MSE(f, T) = \mu ^2(f, T) + \sigma ^2(f, T)$, where

20 $$\begin{aligned} \mu (f, T)= & {} \left| {\mathbb {E}_{x}[\pi _T(f)] - \pi (f)}\right| \quad \text{ and } \quad \sigma ^2(f, T) = \mathbb {E}_{x}\left| {\pi _T(f) - {\mathbb {E}_x\pi _T(f)}}\right| ^2 \nonumber \\= & {} \text{ Var }[\pi _T(f)]. \end{aligned}$$

Here $\mu (f, T)$ measures the bias of the estimator $\pi _T(f)$ and $\sigma ^2(f, T)$ measures the variance of fluctuations of $\pi _T(f)$ around the mean.

The speed of convergence to equilibrium of the process $(X_t)_{t\ge 0}$ will control both the bias term $\mu (f, T)$ and the variance $\sigma ^2(f, T)$. To make this claim more precise, suppose that the semigroup $(P_t)_{t\ge 0}$ associated with $(X_t)_{t\ge 0}$ decays exponentially fast in $L^2(\pi )$, i.e. there exist constants $\lambda > 0$ and $C\ge 1$ such that

21 $$\begin{aligned} \left||P_t g - \pi (g) \right||_{L^2(\pi )} \le C e^{- \lambda t} \left||g-\pi (g) \right||_{L^2(\pi )},\quad g\in L^2(\pi ). \end{aligned}$$

#### Remark 2

If (21) holds with $C=1$, this estimate is equivalent to $-{{\mathrm{\mathcal {L}}}}$ having a spectral gap in $L^2(\pi )$. Allowing for a constant $C>1$ is essential for our purposes though in order to treat nonreversible and degenerate diffusion processes by the theory of *hypocoercivity* as outlined in [54].

The following lemma characterises the decay of the bias $\mu (f,T)$ as $T\rightarrow \infty$ in terms of $\lambda$ and *C*. The proof can be found in [41].

#### Lemma 1

Let $(X_t)_{t\ge 0}$ be the unique, non-explosive solution of (9), such that $X_0 \sim \pi _0 \ll \pi$ and $\frac{d\pi _0}{d\pi } \in L^2(\pi )$, where $\frac{d\pi _0}{d\pi }$ denotes the Radon-Nikodym derivative of $\pi _0$ with respect to $\pi$. Suppose that the process is ergodic with respect to $\pi$ such that the Markov semigroup $(P_t)_{t\ge 0}$ satisfies (21). Then for $f \in L^\infty (\pi )$,

$$\begin{aligned} \mu (f, T) \le \frac{C}{\lambda T}\left( {1 - e^{-\lambda T}}\right) ||f ||_{L^\infty }\text{ Var }_{\pi }\left[ \frac{d\pi _0}{d\pi }\right] ^{\frac{1}{2}}. \end{aligned}$$

The study of the long time behaviour of the variance $\sigma ^2(f, T)$ involves deriving a central limit theorem for the additive functional $\int _0^t f(X_t)-\pi (f)\,\mathrm {d}t$. As discussed in [13], we reduce this problem to proving well-posedness of the Poisson equation

22 $$\begin{aligned} -{{\mathrm{\mathcal {L}}}}\chi = f - \pi (f),\quad \pi (\chi ) = 0. \end{aligned}$$

The only complications in this approach arise from the fact that the generator ${{\mathrm{\mathcal {L}}}}$ need not be symmetric in $L^2(\pi )$ nor uniformly elliptic. The following result summarises conditions for the well-posedness of the Poisson equation and it also provides with us with a formula for the asymptotic variance. Again, the proof can be found in [41].

#### Lemma 2

Let $(X_t)_{t\ge 0}$ be the unique, non-explosive solution of (9) with smooth drift and diffusion coefficients, such that the corresponding infinitesimal generator is hypoelliptic. Syppose that $(X_t)_{t\ge 0}$ is ergodic with respect to $\pi$ and moreover, $(P_t)_{t\ge 0}$ decays exponentially fast in $L^2(\pi )$ as in (21). Then for all $f\in L^2(\pi )$, there exists a unique mean zero solution $\chi$ to the Poisson equation (22). If $X_0 \sim \pi$, then for all $f \in C^\infty (\mathbb {R}^d) \cap L^2(\pi )$

23 $$\begin{aligned} \sqrt{T}\left( \pi _T(f) - \pi (f)\right) \xrightarrow [T\rightarrow \infty ]{d} \mathcal {N}(0, 2\sigma ^2_f), \end{aligned}$$

where $\sigma ^2_f$ is the asymptotic variance defined by

24 $$\begin{aligned} \sigma ^2_{f} = \left\langle \chi , (-{{\mathrm{\mathcal {L}}}})\chi \right\rangle _{L^2(\pi )} = \left\langle \nabla \chi , \varSigma \nabla \chi \right\rangle _{L^2(\pi )}. \end{aligned}$$

Moreover, if $X_0 \sim \pi _0$ where $\pi _0 \ll \pi$ and $\frac{d\pi _0}{d\pi }\in L^2(\pi )$ then (23) holds for all $f \in C^\infty (\mathbb {R}^d) \cap {L^\infty (\mathbb {R}^d)}$.^1^

Clearly, observables that only differ by a constant have the same asymptotic variance. In the sequel, we will hence restrict our attention to observables $f\in L^{2}(\pi )$ satisfying $\pi (f)=0$, simplifying expressions (22) and (23). The corresponding subspace of $L^2(\pi )$ will be denoted by $L_{0}^2(\pi )$. If the exponential decay estimate (21) is satisfied, then Lemma 2 shows that $-{{\mathrm{\mathcal {L}}}}$ is invertible on $L^2_{0}(\pi )$, so we can express the asymptoptic variance as

25 $$\begin{aligned} \sigma _{f}^2=\langle f, (-{{\mathrm{\mathcal {L}}}})^{-1} f \rangle _{L^2(\pi )}, \quad f \in L^2_{0}(\pi ). \end{aligned}$$

We note that the constants *C* and $\lambda$ appearing in the exponential decay estimate (21) also control the speed of convergence of $\sigma ^2(f, T)$ to zero. Indeed, it is straightforward to show that if (21) is satisfied, then the solution $\chi$ of (22) satisfies

26 $$\begin{aligned} \sigma ^2_{f} = \left\langle \chi , f-\pi (f) \right\rangle _{L^2(\pi )} \le \frac{C}{\lambda }\left||{f}\right||^2_{L^2(\pi )}. \end{aligned}$$

Lemmas 1 and 2 would suggest that choosing the coefficients $\varSigma$ and $\gamma$ to optimize the constants *C* and $\lambda$ in (26) would be an effective means of improving the performance of the estimator $\pi _T(f)$, especially since the improvement in performance would be uniform over an entire class of observables. When this is possible, this is indeed the case. However, as has been observed in [20, 21, 34], maximising the speed of convergence to equilibrium is a delicate task. As the leading order term in *MSE*(*f*, *T*), it is typically sufficient to focus specifically on the asymptotic variance $\sigma ^2_{f}$ and study how the parameters of the SDE (9) can be chosen to minimise $\sigma ^2_{f}$. This study was undertaken in [14] for processes of the form (7).

## Perturbation of Underdamped Langevin Dynamics

The primary objective of this work is to compare the performances of the perturbed underdamped Langevin dynamics (8) and the unperturbed dynamics (4) according to the criteria outlined in Sect. 2.3 and to find suitable choices for the matrices $J_{1}$, $J_{2}$, *M* and $\varGamma$ that improve the performance of the sampler. We begin our investigations of (8) by establishing ergodicity and exponentially fast return to equilibrium, and by studying the overdamped limit of (8). As the latter turns out to be nonreversible and therefore in principle superior to the usual overdamped limit (3), e.g. [21], this calculation provides us with further motivation to study the proposed dynamics.

For the bulk of this work, we focus on the particular case when the target measure is Gaussian, i.e. when the potential is given by $V(q)=\frac{1}{2}q^{T}Sq$ with a symmetric and positive definite precision matrix *S* (i.e. the covariance matrix is given by $S^{-1}$). In this case, we advocate the following conditions for the choice of parameters:

27 $$\begin{aligned} M =S,\quad \varGamma =\gamma S, \quad SJ_{1}S =J_{2},\quad \mu =\nu . \end{aligned}$$

Under the above choices (27), we show that the large perturbation limit $\lim _{\mu \rightarrow \infty } \sigma _f^2$ exists and is finite and we provide an explicit expression for it (see Corollary 4). From this expression, we derive an algorithm for finding optimal choices for $J_1$ in the case of quadratic observables (see Algorithm 2).

If the friction coefficient is not too small ($\gamma > \sqrt{2}$), and under certain mild nondegeneracy conditions, we prove that adding a small perturbation will always decrease the asymptotic variance for observables of the form $f(q)=q\cdot Kq+l\cdot q+C$:

$$\begin{aligned} \left. \frac{\mathrm {d}}{\mathrm {d}\mu }\sigma _{f}^{2}\right| _{\mu =0}=0\quad \text {and }\quad \left. \frac{\mathrm {d}^{2}}{\mathrm {d}\mu ^{2}}\sigma _{f}^{2}\right| _{\mu =0}<0, \end{aligned}$$

see Theorem 3. In fact, we conjecture that this statement is true for arbitrary observables $f\in L^{2}(\pi )$, but we have not been able to prove this. The dynamics (8) [used in conjunction with the conditions (27)] proves to be especially effective when the observable is antisymmetric (i.e. when it is invariant under the substitution $q\mapsto -q$) or when it has a significant antisymmetric part. In particular, in Proposition 3 we show that under certain conditions on the spectrum of $J_1$, for any antisymmetric observable $f\in L^{2}(\pi )$ it holds that $\lim _{\mu \rightarrow \infty }\sigma _{f}^{2}=0$.

Numerical experiments and analysis show that departing significantly from (27) in fact possibly decreases the performance of the sampler. This is in stark contrast to (7), where it is not possible to increase the asymptotic variance by *any* perturbation. For that reason, until now it seems practical to use (8) as a sampler only when a reasonable estimate of the global covariance of the target distribution is available. In the case of Bayesian inverse problems and diffusion bridge sampling, the target measure $\pi$ is given with respect to a Gaussian prior. We demonstrate the effectiveness of our approach in these applications, taking the prior Gaussian covariance as *S* in (27).

### Remark 3

In [34, Rem. 3] another modification of (4) was suggested (albeit with the simplifications $\varGamma =\gamma \cdot I$ and $M=I$):

28 $$\begin{aligned} \mathrm {d}q_{t} =(1-J)M^{-1}p_{t}\mathrm {d}t ,\quad \mathrm {d}p_{t} =-(1+J)\nabla V(q_{t})\mathrm {d}t-\varGamma M^{-1}p_{t}\mathrm {d}t+\sqrt{2\varGamma }\mathrm {d}W_{t}, \end{aligned}$$

*J* again denoting an antisymmetric matrix. However, under the change of variables $p\mapsto (1+J)\tilde{p}$ the above equations transform into

29 $$\begin{aligned} \mathrm {d}q_{t} =\tilde{M}^{-1}p_{t}\mathrm {d}t,\quad \mathrm {d}\tilde{p}_{t} =-\nabla V(q_{t})\mathrm {d}t-\tilde{\varGamma }\tilde{M}^{-1}\tilde{p}_{t}\mathrm {d}t+\sqrt{2\tilde{\varGamma }}\mathrm {d}\tilde{W}_{t}, \end{aligned}$$

where $\tilde{M}=(1+J)^{-1}M(1-J)^{-1}$ and $\tilde{\varGamma }=(1+J)^{-1}\varGamma (1-J)^{-1}$. Since any observable *f* depends only on *q* (the *p*-variables are merely auxiliary), the estimator $\pi _T(f)$ as well as its associated convergence characteristics (i.e. asymptotic variance and speed of convergence to equilibrium) are invariant under this transformation. Therefore, (28) reduces to the underdamped Langevin dynamics (4) and does not represent an independent approach to sampling. Suitable choices of *M* and $\varGamma$ will be discussed in Sect. 4.5.

### Properties of Perturbed Underdamped Langevin Dynamics

In this section we study some of the properties of the perturbed underdamped dynamics (8). First, note that its generator is given by

30 $$\begin{aligned} \mathcal {L}=\underbrace{\underbrace{M^{-1}p\cdot \nabla _{q}-\nabla _{q}V\cdot \nabla _{p}}_{\mathcal {L}_{ham}}\underbrace{-\varGamma M^{-1}p\cdot \nabla _{p}+\varGamma : D^2_{p}}_{\mathcal {L}_{therm}}}_{\mathcal {L}_0} \underbrace{-\mu J_{1}\nabla V \cdot \nabla _{q} - \nu J M^{-1} p \cdot \nabla _{p}}_{\mathcal {L}_{pert}}, \end{aligned}$$

decomposed into the perturbation $\mathcal {L}_{pert}$ and the unperturbed operator $\mathcal {L}_0$, which can be further split into the Hamiltonian part $\mathcal {L}_{ham}$ and the thermostat (Ornstein–Uhlenbeck) part $\mathcal {L}_{therm}$, see [35, 36, 46].

#### Lemma 3

The infinitesimal generator ${{\mathrm{\mathcal {L}}}}$ (30) is hypoelliptic.

#### Proof

The proof consists of verifying the conditions of Hörmander’s Theorem for the generator (30) and can be found in [41]. $\square$

An immediate corollary of this result and of Theorem 1 is that the perturbed underdamped Langevin process (8) is ergodic with unique invariant distribution $\widehat{\pi }$ given by (5).

As explained in Sect. 2.3, the exponential decay estimate (21) is crucial for our approach, as in particular it guarantees the well-posedness of the Poisson equation (22). From now on, we will therefore make the following assumption on the potential *V*,  required to prove exponential decay in $L^2(\pi )$:

#### Assumption 1

Assume that the Hessian of *V* is *bounded* and that the target measure $\pi (\mathrm {d}q) = \frac{1}{Z}e^{-V}\mathrm {d}q$ satisfies a *Poincare inequality*, i.e. there exists a constant $\rho >0$ such that

31 $$\begin{aligned} \int _{\mathbb {R}^d}\phi ^2\mathrm {d}\pi \le \rho \int _{\mathbb {R}^d} \vert \nabla \phi \vert ^2 \mathrm {d}\pi , \quad \phi \in L_{0}^2(\pi )\cap H^1(\pi ). \end{aligned}$$

Sufficient conditions on the potential so that Poincaré’s inequality holds, e.g. the Bakry-Emery criterion, are presented in [7].

#### Theorem 2

Under Assumption 1 there exist constants $C\ge 1$ and $\lambda >0$ such that the semigroup $(P_t)_{t\ge 0}$ generated by ${{\mathrm{\mathcal {L}}}}$ satisfies exponential decay in $L^2(\pi )$ as in (21).

#### Proof

The proof uses the machinery of hypocoercivity developed in [54] and can be found in [41]. Using the framework of [15], we conjecture that the assumption on the boundedness of the Hessian of *V* can be substantially weakened and more quantitative decay estimates (in particular with respect to $\mu$ and $\nu$) can be obtained. This approach has recently been successfully applied to equilibrium and nonequilibirum Langevin dynamics, see [27, 53]. We leave this work track for future study. $\square$

### The Overdamped Limit

In this section we develop a connection between the perturbed underdamped Langevin dynamics (8) and the nonreversible overdamped Langevin dynamics (7). The analysis is very similar to the one presented in [35, Sect. 2.2.2] and we will be brief. For convenience in this section we will perform the analysis on the *d*-dimensional torus $\mathbb {T}^d \cong (\mathbb {R} / \mathbb {Z})^d$, i.e. we will assume $q \in \mathbb {T}^d$. Consider the following scaling of (8):

32a $$\begin{aligned} \mathrm {d}q_{t}^{\epsilon }= & {} \frac{1}{\epsilon }M^{-1}p_{t}^{\epsilon },\mathrm {d}t-\mu J_{1}\nabla _{q}V(q_{t})\mathrm {d}t, \end{aligned}$$

32b $$\begin{aligned} \mathrm {d}p_{t}^{\epsilon }= & {} -\frac{1}{\epsilon }\nabla _{q}V(q_{t}^{\epsilon })\mathrm {d}t-\frac{1}{\epsilon ^{2}}\nu J_{2}M^{-1}p_{t}^{\epsilon }\mathrm {d}t-\frac{1}{\epsilon ^{2}}\varGamma M^{-1}p_{t}^{\epsilon }\mathrm {d}t+\frac{1}{\epsilon }\sqrt{2\varGamma }\mathrm {d}W_{t}, \end{aligned}$$

valid for the small mass/small momentum regime $M \rightarrow \epsilon ^{2}M, \, p_{t} \rightarrow \epsilon p_{t}.$ Equivalently, those modifications can be obtained from subsituting $\varGamma \rightarrow \epsilon ^{-1}\varGamma$ and $t\mapsto \epsilon ^{-1}t$, and so in the limit as $\epsilon \rightarrow 0$ the dynamics (32) describes the limit of large friction with rescaled time. It turns out that as $\epsilon \rightarrow 0$, the dynamics (32) converges to the limiting SDE

33 $$\begin{aligned} \mathrm {d}q_{t}=-(\nu J_{2}+\varGamma )^{-1}\nabla _{q}V(q_{t})\mathrm {d}t-\mu J_{1}\nabla _{q}V(q_{t})\mathrm {d}t+(\nu J_{2}+\varGamma )^{-1}\sqrt{2\varGamma }\mathrm {d}W_{t}. \end{aligned}$$

The following proposition makes this statement precise.

#### Proposition 1

Denote by $(q_{t}^{\epsilon },p_{t}^{\epsilon })$ the solution to (32) with (deterministic) initial conditions $(q_{0}^{\epsilon },p_{0}^{\epsilon })=(q_{init},p_{init})$ and by $q_{t}^{0}$ the solution to (33) with initial condition $q_{0}^{0}=q_{init}.$ For any $T>0$, $(q_{t}^{\epsilon })_{0\le t\le T}$ converges to $(q_{t}^{0})_{0\le t\le T}$ in $L^{2}(\Omega ,C([0,T]),\mathbb {T}^{d})$ as $\epsilon \rightarrow 0$, i.e. $\lim _{\epsilon \rightarrow 0}\mathbb {E}\big (\sup _{0\le t\le T}\vert q_{t}^{\epsilon }-q_{t}^{0}\vert ^{2}\big )=0.$

#### Proof

The proof follows standard arguments (see for instance [46]) and can be found in [41]. By a more refined analysis, it is also possible to get information on the rate of convergence; see, e.g. [48, 49]. $\square$

#### Remark 4

The overdamped limit (33) respects the invariant distribution, in the sense that it is ergodic with respect to $\pi (\mathrm {d}q) = \frac{1}{Z}e^{-V}\mathrm {d}q$.

The limiting SDE (33) is nonreversible due to the term $-\mu J_1 \nabla _q V(q_t)\mathrm {d}t$ and also because the matrix $(\nu J_{2}+\varGamma )^{-1}$ is in general neither symmetric nor antisymmetric. This result, together with the fact that nonreversible perturbations of overdamped Langevin dynamics of the form (7) are by now well-known to have improved performance properties, motivates further investigation of the dynamics (8).

## Sampling from a Gaussian Distribution

In this section we study in detail the performance of the Langevin sampler (8) for Gaussian target densities, first considering the case of unit covariance. In particular, we study the optimal choice for the parameters in the sampler, the exponential decay rate and the asymptotic variance. We then extend our results to Gaussian target densities with arbitrary covariance matrices.

### Unit Covariance: Small Perturbations

In our study of the dynamics given by (8) we first consider the simple case when $V(q)=\frac{1}{2}\vert q\vert ^{2}$, i.e. the task of sampling from a Gaussian measure with unit covariance. We will assume $M=I$, $\varGamma =\gamma I$ and $J_{1}=J_{2}=:J$ (so that the $q-$ and $p-$dynamics are perturbed in the same way, albeit posssibly with different strengths $\mu$ and $\nu$). Our first result concerns the asymptotic variance for linear and quadratic observables for small perturbations of equal strength ($\mu = \nu$). For sufficiently strong damping $(\gamma >\sqrt{2}$) always leads to an improvement in asymptotic variance under the nondegeneracy conditions $[J,K]\ne 0$ and $l\notin \ker J$:

#### Theorem 3

Consider the dynamics

34 $$\begin{aligned} \mathrm {d}q_{t}= & {} p_{t}\mathrm {d}t-\mu Jq_{t}\mathrm {d}t, \nonumber \\ \mathrm {d}p_{t}= & {} -q_{t}\mathrm {d}t-\mu Jp_{t}\mathrm {d}t-\gamma p_{t}\mathrm {d}t+\sqrt{2\gamma }\mathrm {d}W_{t}, \end{aligned}$$

with $\gamma >\sqrt{2}$ and an observable of the form $f(q)=q\cdot Kq+l\cdot q+C$, where $K\in \mathbb {R}_{sym}^{d\times d}$, $l\in \mathbb {R}^{d}$ and $C\in \mathbb {R}$. If at least one of the conditions $[J,K]\ne 0$ and $l\notin \ker J$ is satisfied, then the asymptotic variance of the unperturbed sampler is at a local maximum independently of *K* and *J* (and $\gamma$, as long as $\gamma >\sqrt{2}$), i.e.

$$\begin{aligned} \left. \partial _{\mu }\sigma _{f}^{2} \right| _{\mu =0}=0 \quad \text{ and }\quad \left. \partial _{\mu }^{2}\sigma _{f}^{2}\right| _{\mu = 0}<0. \end{aligned}$$

#### Proof

The dynamics (34) are of Ornstein–Uhlenbeck type, i.e. we can write

35 $$\begin{aligned} \mathrm {d}X_{t}=-BX_{t}\mathrm {d}t+\sqrt{2Q}\mathrm {d}\bar{W}_{t},\quad B=\left( \begin{array}{c@{\quad }c} \mu J &{} -I\\ I &{} \gamma I+\mu J \end{array}\right) , \quad Q=\left( \begin{array}{c@{\quad }c} \varvec{0} &{} \varvec{0}\\ \varvec{0} &{} \gamma I \end{array}\right) \end{aligned}$$

with $X=(q,p)^{T}$, and $(\bar{W}_{t})_{t\ge 0}$ denoting a standard Wiener process on $\mathbb {R}^{2d}$. The generator of (35) is then given by

36 $$\begin{aligned} \mathcal {L}=-Bx\cdot \nabla +\nabla ^{T}Q\nabla . \end{aligned}$$

According to Lemma 2, the asymptotic variance can be expressed as

37 $$\begin{aligned} \sigma _{f}^{2}=\langle \chi ,f\rangle _{L^{2}(\widehat{\pi })},\quad \text{ where } \chi \text{ is } \text{ the } \text{ solution } \text{ to } \quad -\mathcal {L}\chi =f,\quad \widehat{\pi }(\chi )=0. \end{aligned}$$

By calculations similar to those in [14, Sect. 4], $\chi$ is given by $\chi (x)=x\cdot Cx+D\cdot x-{{\mathrm{Tr}}}C$, where

38 $$\begin{aligned} BC+CB^{T} =\bar{K}, \quad B^{T}D =\bar{l}, \end{aligned}$$

using the notations

39 $$\begin{aligned} \bar{K}=\left( \begin{array}{c@{\quad }c} K &{} \varvec{0}\\ \varvec{0} &{} \varvec{0} \end{array}\right) \in \mathbb {R}^{2d\times 2d} \quad \text{ and }\quad \bar{l}=\left( \begin{array}{c} l\\ \varvec{0} \end{array}\right) \in \mathbb {R}^{2d}. \end{aligned}$$

The asymptotic variance is then given by

40 $$\begin{aligned} \sigma _{f}^{2}=2{{\mathrm{Tr}}}(C\bar{K})+D\cdot \bar{l}. \end{aligned}$$

Taking derivatives of 38 and solving the ensuing matrix equations, it is possible to obtain explicit expressions for $\partial _{\mu }C\vert _{\mu =0}$, $\partial ^{2}_{\mu }C\vert _{\mu =0}$, $\partial _{\mu }D\vert _{\mu =0}$ and $\partial ^{2}_{\mu }C\vert _{\mu =0}$ as detailed in [41]. We obtain

$$\begin{aligned} \left. \partial _{\mu }\sigma _{f}^{2} \right| _{\mu =0}= & {} 0 \quad \text{ and }\quad \left. \partial _{\mu }^{2}\sigma _{f}^{2}\right| _{\mu = 0}=(-2\gamma ^3+4\gamma )\vert Jl \vert ^{2}\\&+ \left( \gamma - \frac{4}{\gamma ^3} - \gamma ^3 - \frac{1}{\gamma }\right) \cdot \left( {{\mathrm{Tr}}}(JKJK)-{{\mathrm{Tr}}}(J^2 K^2)\right) . \end{aligned}$$

Notice that ${{\mathrm{Tr}}}(JKJK)-{{\mathrm{Tr}}}(J^{2}K^{2})=\frac{1}{2}{{\mathrm{Tr}}}([J,K]^{2})$ and that [*J*, *K*] is symmetric. It follows that ${{\mathrm{Tr}}}(JKJK)-{{\mathrm{Tr}}}(J^{2}K^{2})\ge 0$ with equality if and only if $[J,K]=0$. Together with $-2\gamma ^3+4\gamma < 0$ for $\gamma > \sqrt{2}$ and $\gamma - \frac{4}{\gamma ^3} - \gamma ^3 - \frac{1}{\gamma } < 0$ for $\gamma >0$, the claim follows. $\square$

#### Remark 5

As we will see in Sect. 4.3, Example 1, if $[J,K]=0$ and $l\in \ker J$, the asymptotic variance is constant as a function of $\mu$, i.e. the perturbation has no effect.

Numerical examples show that the conditions $\gamma >\sqrt{2}$ and $\mu =\nu$ are indeed necessary for the conclusions of Theorem 3 to hold (an explanation in terms of the spectrum of the generator will be provided in Sect. 4.2). In particular, an unfortunate choice of the perturbations will actually increase the asymptotic variance of the dynamics.

**Fig. 1:**
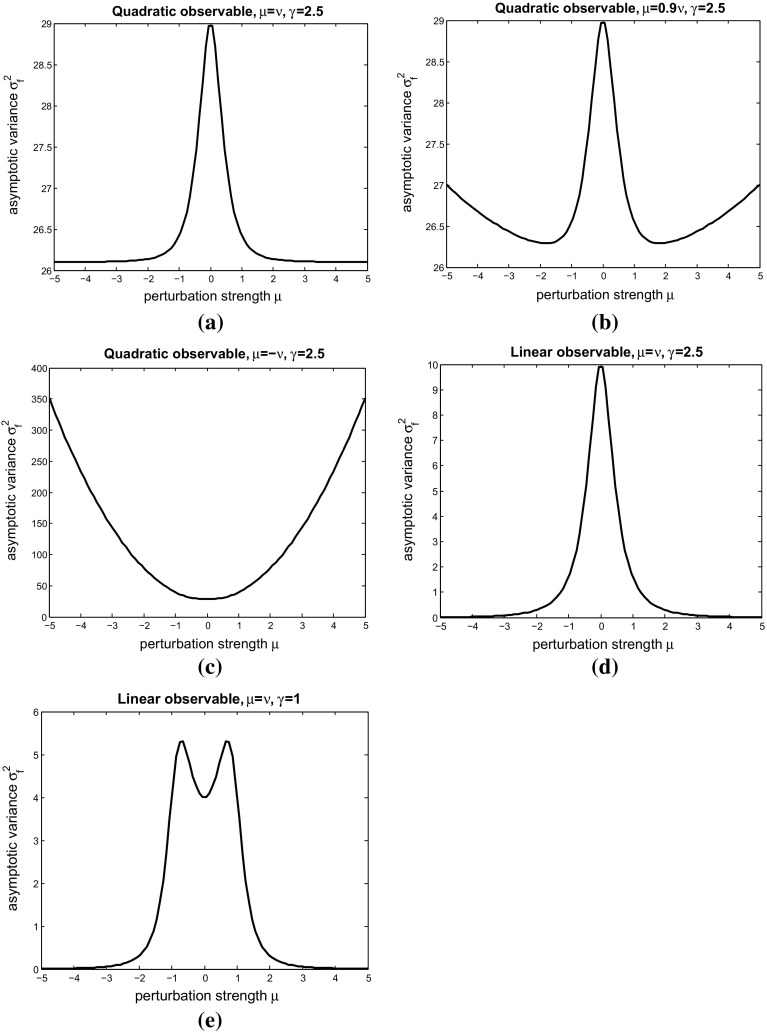
Asymptotic variance for linear and quadratic observables, depending on relative perturbation and friction strengths. **a** Equal perturbations: $\mu =\nu$. **b** Approximately equal perturbations: $\mu =0.9\nu$. **c** Opposing perturbations: $\mu =-\nu$. **d** Equal perturbations: $\mu =\nu$ (sufficiently large friction $\gamma$). **e** Equal perturbations: $\mu =\nu$ (small friction $\gamma$)

Let us illustrate this by plotting the asymptotic variance as a function of the perturbation strength $\mu$ (see Fig. 1), making the choices $d=2$, $l=(1,1)^{T}$,

41 $$\begin{aligned} K=\left( \begin{array}{c@{\quad }c} 2 &{} 0\\ 0 &{} 1 \end{array}\right) \quad \text {and} \quad J=\left( \begin{array}{c@{\quad }c} 0 &{} 1\\ -1 &{} 0 \end{array}\right) . \end{aligned}$$

The asymptotic variance has been computed according to (38) and (40). Going beyond the results of this section, the graphs give an impression of the behaviour of the asymptotic variace for large values of $\mu$, discussed further in Sect. 4.3.

Figure 1a, b, c show the asymptotic variance associated with the quadratic observable $f(q)=q\cdot K q$. In accordance with Theorem 3, the asymptotic variance is at a local maximum at zero perturbation in the case $\mu =\nu$ (see Fig. 1a). For increasing perturbation strength, the graph shows monotone decay for $\mu \rightarrow \infty$ (this limiting behaviour will be explored analytically in Sect. 4.3). If the condition $\mu =\nu$ is only approximately satisfied (Fig. 1b), our numerical examples still exhibits decaying asymptotic variance in the neighbourhood of the critical point. In this case, however, the asymptotic variance diverges for growing values of the perturbation $\mu$. If the perturbations are opposed ($\mu =-\nu$), it is possible for certain observables that the unperturbed dynamics represents a global minimum. Such a case is observed in Fig. 1c. In Fig. 1d, e the observable $f(q)=l\cdot q$ is considered. If the damping is sufficiently strong ($\gamma > \sqrt{2}$), the unperturbed dynamics is at a local maximum of the asymptotic variance (Fig. 1d). Furthermore, the asymptotic variance approaches zero as $\mu \rightarrow \infty$ (for a theoretical explanation see again Sect. 4.3). The graph in Fig. 1e shows that the assumption of $\gamma$ not being too small cannot be dropped from Theorem 3. Even in this case though the example shows decay of the asymptotic variance for large values of $\mu$.

### Exponential Decay Rate

Let us denote by $\lambda ^{*}$ the *optimal exponential decay rate* in (21), i.e.

42 $$\begin{aligned} \lambda ^{*}=\sup \{\lambda > 0 \, \vert \, \text {There exists } C\ge 1 \text { such that } (21) \text { holds}\}. \end{aligned}$$

Note that $\lambda ^{*}$ is well-defined and positive by Theorem 2. We also define the *spectral bound* of the generator ${{\mathrm{\mathcal {L}}}}$ by

43 $$\begin{aligned} s({{\mathrm{\mathcal {L}}}})=\inf (\text {Re}\,\sigma (-{{\mathrm{\mathcal {L}}}})\setminus \{0\}). \end{aligned}$$

In [38] it is proven that the Ornstein–Uhlenbeck semigroup $(P_t)_{t\ge 0}$ considered in this section is differentiable (see Proposition 2.1). In this case (see Corollary 3.12 of [17]), it is known that the exponential decay rate and the spectral bound coincide, i.e. $\lambda ^{*}=s({{\mathrm{\mathcal {L}}}})$, whereas in general only $\lambda ^{*}\le s({{\mathrm{\mathcal {L}}}})$ holds. In this section we will therefore analyse the spectral properties of the generator $\mathcal {L}$ in the Gaussian case. In particular, this leads to some intuition of why choosing equal perturbations ($\mu =\nu$) is crucial for the performance of the sampler.

In [38] (see also [43]), it was proven that the spectrum of $\mathcal {L}$ as in (36) in $L^{2}(\widehat{\pi })$ is given by

44 $$\begin{aligned} \sigma (\mathcal {L})=\left\{ -\sum _{j=1}^{r}n_{j}\lambda _{j}:\, n_{j}\in \mathbb {N},\lambda _{j}\in \sigma (B)\right\} . \end{aligned}$$

Note that $\sigma (\mathcal {L})$ only depends on the drift matrix *B*. In the case where $\mu =\nu$, the spectrum of *B* can be computed explicitly.

#### Lemma 4

Assume $\mu =\nu$. Then the spectrum of *B* is given by^2^

45 $$\begin{aligned} \sigma (B)=\left\{ \mu \lambda +\sqrt{\big (\frac{\gamma }{2}\big )^{2}-1}+\frac{\gamma }{2}\vert \lambda \in \sigma (J)\right\} \cup \left\{ \mu \lambda -\sqrt{\big (\frac{\gamma }{2}\big )^{2}-1}+\frac{\gamma }{2}\vert \lambda \in \sigma (J)\right\} . \end{aligned}$$

#### Proof

We will compute $\sigma \big (B-\frac{\gamma }{2}I\big )$ and then use the identity $\sigma (B)=\left\{ \lambda +\frac{\gamma }{2}\vert \lambda \in \sigma \left( B-\frac{\gamma }{2}I\right) \right\} .$ We have

$$\begin{aligned} \det \left( B-\frac{\gamma }{2}I-\lambda I\right)&=\det \left( \left( \mu J-\frac{\gamma }{2}I-\lambda I\right) \left( \mu J+\frac{\gamma }{2}I-\lambda I\right) +I\right) \\&=\det \left( \mu J-\lambda I+\sqrt{\left( \frac{\gamma }{2}\right) ^{2}-1} I\right) \cdot \det \left( \mu J-\lambda I-\sqrt{\left( \frac{\gamma }{2}\right) ^{2}-1} I\right) , \end{aligned}$$

where *I* is understood to denote the identity matrix of appropriate dimension. The above quantity is zero if and only if

$$\begin{aligned} \lambda -\sqrt{\left( \frac{\gamma }{2}\right) ^{2}-1}\in \sigma (\mu J) \quad \text{ or }\quad \lambda +\sqrt{\left( \frac{\gamma }{2}\right) ^{2}-1}\in \sigma (\mu J). \end{aligned}$$

Together with (4.2), the claim follows. $\square$

**Fig. 2:**
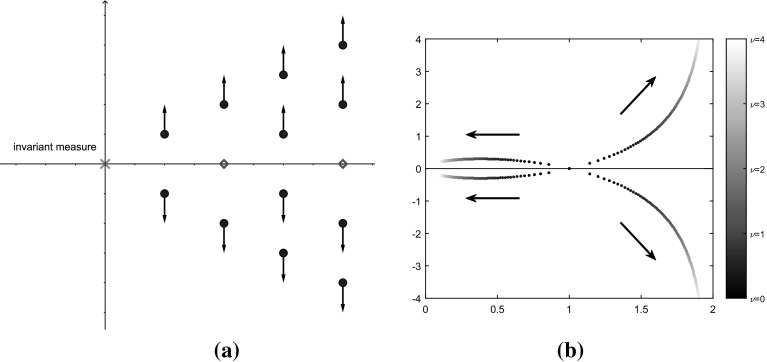
Effects of the perturbation on the spectra of $-{{\mathrm{\mathcal {L}}}}$ and *B*. **a** $\sigma (-{{\mathrm{\mathcal {L}}}})$ in the case $\mu =\nu$. The arrows indicate the movement of the spectrum as the perturbation strength $\mu$ increases. **b** $\sigma (B)$ in the case $J_{1}=0$, i.e. the dynamics is only perturbed by $-\nu J_{2}p\mathrm {d}t$. The arrows indicate the movement of the eigenvalues as $\nu$ increases

Using formula (45), in Fig. 2a we show a sketch of the spectrum $\sigma (-\mathcal {L}$) for the case of equal perturbations ($\mu =\nu )$ with the convenient choices $n=1$ and $\gamma =2.$ Of course, the eigenvalue at 0 is associated to the invariant measure since $\mathcal {L}^{\dagger }\widehat{\pi }=0$. The arrows indicate the movement of the eigenvalues as the perturbation $\mu$ increases in accordance with Lemma 4. Clearly, the spectral bound of ${{\mathrm{\mathcal {L}}}}$ is not affected by the perturbation. Note that the eigenvalues on the real axis stay invariant under the perturbation. The subspace of $L_{0}^{2}(\widehat{\pi })$ associated to those will turn out to be crucial for the characterisation of the limiting asymptotic variance as $\mu \rightarrow \infty$ (see Remark 10).

To illustrate the suboptimal properties of the perturbed dynamics when the perturbations are not equal, we plot the spectrum of the drift matrix $\sigma (B)$ in the case when the dynamics is only perturbed by the term $\nu J_{2}p\mathrm {d}t$ (i.e. $\mu =0$) for $n=2$, $\gamma =2$ and

46 $$\begin{aligned} J_2=\left( \begin{array}{c@{\quad }c} 0 &{} -1\\ 1 &{} 0 \end{array}\right) , \end{aligned}$$

(see Fig. 2b). Note that the full spectrum $\sigma (-\mathcal {L})$ can be inferred from (44). For $\nu =0$ we have that the spectrum $\sigma (B)$ only consists of the (degenerate) eigenvalue 1. For increasing $\nu$, the figure shows that the degenerate eigenvalue splits up into four eigenvalues, two of which get closer to the imaginary axis as $\nu$ increases, leading to a smaller spectral bound and therefore to a decrease in the speed of convergence to equilibrium. Figure 2a, b give an intuitive explanation of why the fine-tuning of the perturbation strengths is crucial.

**Fig. 3:**
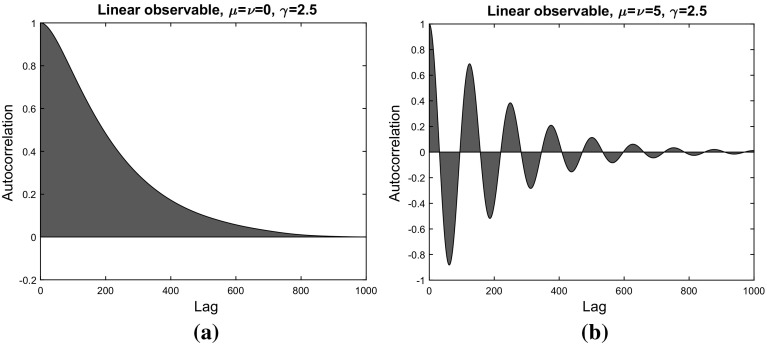
Autocorrelation plots for the perturbed and unperturbed dynamics. **a** Unperturbed Langevin dynamics. **b** Perturbed Langevin dynamics

We close this subsection by providing autocorrelation plots (see Fig. 3) for the linear observable considered in Fig. 1d (with a friction coefficient of $\gamma = 2.5$). It is well-known that the asymptotic variance is given by the integrated autocorrelation function (see e.g. Proposition IV 1.3 in [3]),

47 $$\begin{aligned} \sigma _f^2 = \int _0^{\infty } \mathbb {E}_{\pi } \left[ \left( f(q_0) - \pi (f)\right) \left( f(q_t) - \pi (f) \right) \right] \mathrm {d}t. \end{aligned}$$

Comparing Fig. 3a, b yields additional insight into the mechanics of the variance reduction: the increase of the imaginary part of the eigenvalues of $\mathcal {L}$ (as indicated in Fig. 2a) leads to oscillations in the autocorrelation function and therefore to cancellations in (47). A similar effect has already been observed in [50] for the nonreversible overdamped Langevin dynamics (15).

### Unit Covariance: Large Perturbations

In the previous subsection we observed that for the particular perturbation $J_1 = J_2$ and $\mu = \nu$ [see equation (34)] the perturbed Langevin dynamics demonstrated an improvement in performance for $\mu$ in a neighbourhood of 0, when the observable is linear or quadratic. Recall that this dynamics is ergodic with respect to a standard Gaussian measure $\hat{\pi }$ on $\mathbb {R}^{2d}$ with marginal $\pi$ with respect to the *q*-variable. As before, we shall consider only observables that do not depend on *p*. Moreover, we assume without loss of generality that $\pi (f) = 0$. For such observables we will write $f \in L_0^2(\pi )$ and consider the canonical embedding $L_0^2(\pi ) \subset L^2(\hat{\pi })$. We emphasize that $L_0^2(\pi )$ consists of functions that only depend on *q*, whereas functions in $L^2(\hat{\pi })$ may depend on both *q* and *p*.

In this subsection will analyse the asymptotic variance for large values of $\mu$. The infinitesimal generator of (34) can be written as

48 $$\begin{aligned} \mathcal {L}=\underbrace{p\cdot \nabla _{q}-q\cdot \nabla _{p}+\gamma (-p\cdot \nabla _{p}+\varDelta _{p})}_{\mathcal {L}_{0}}+\mu \underbrace{(-Jq\cdot \nabla _{q}-Jp\cdot \nabla _{p})}_{\mathcal {A}}=:\mathcal {L}_{0}+\mu \mathcal {A}, \end{aligned}$$

where we have introduced the notation $\mathcal {L}_{pert}=\mu \mathcal {A}$. In the sequel, the adjoint of an operator $\mathcal {B}$ in $L^2(\widehat{\pi })$ will be denoted by $\mathcal {B}^{*}$. In the rest of this section we will make repeated use of the Hermite polynomials

49 $$\begin{aligned} g_{\alpha }(x)=(-1)^{\vert \alpha \vert }e^{\frac{\vert x\vert ^{2}}{2}}\nabla ^{\alpha }e^{-\frac{\vert x\vert ^{2}}{2}},\quad \alpha \in \mathbb {N}^{2d}, \end{aligned}$$

invoking the notation $x=(q,p)\in \mathbb {R}^{2d}$. For $m\in \mathbb {N}_{0}$ define the Hilbert spaces

$$\begin{aligned} H_{m}={{\mathrm{span}}}\{g_{\alpha }:\,\vert \alpha \vert =m\},\quad \langle f,g\rangle _{m}:=\langle f,g\rangle _{L^2(\widehat{\pi })},\quad f,g\in H_{m}. \end{aligned}$$

The following result (Theorem 4) holds for operators of the form (36) providing an orthogonal decomposition of $L^{2}(\widehat{\pi })$ into invariant subspaces. The drift and diffusion matrices *B* and *Q* are assumed to be such that $\mathcal {L}$ is the generator of an ergodic stochastic process (see [2, Definition 2.1] for precise conditions).

#### Theorem 4

[2, Sect. 5]. The following holds:

- The space $L^{2}(\widehat{\pi })$ has a decomposition into mutually orthogonal subspaces:
  $$\begin{aligned} L^{2}(\widehat{\pi })=\bigoplus _{m\in \mathbb {N}_{0}}H_{m}. \end{aligned}$$
- For all $m\in \mathbb {N}_{0}$, $H_{m}$ is invariant under $\mathcal {L}$ as well as under the semigroup $(e^{t\mathcal {L}})_{t\ge 0}$.
- The spectrum of $\mathcal {L}$ has the following decomposition:
  $$\begin{aligned} \sigma (\mathcal {L})=\bigcup _{m\in \mathbb {N}_{0}}\sigma (\mathcal {L}\vert _{H_{m}}), \quad \sigma (\mathcal {L}\vert _{H_{m}})=\left\{ \sum _{j=1}^{2d}\alpha _{j}\lambda _{j}:\,\vert \alpha \vert =m,\,\lambda _{j}\in \sigma (B)\right\} . \end{aligned}$$

#### Remark 6

Note that by the ergodicity of the dynamics, $\ker \mathcal {L}$ consists of constant functions and so $\ker \mathcal {L}=H_{0}$. Therefore, $L^2_0(\widehat{\pi })$ has the decomposition

$$\begin{aligned} L_{0}^{2}(\widehat{\pi })=L^{2}(\widehat{\pi })/\ker \mathcal {L}=\bigoplus _{m\ge 1}H_{m}. \end{aligned}$$

Our first main result of this section is an expression for the asymptotic variance in terms of the unperturbed operator $\mathcal {L}_{0}$ and the perturbation $\mathcal {A}$:

#### Proposition 2

Let $f\in L_{0}^{2}(\pi )$ (so in particular $f=f(q)$). Then the associated asymptotic variance is given by

50 $$\begin{aligned} \sigma _{f}^{2}=\langle f,-\mathcal {L}_{0}(\mathcal {L}_{0}^{2}+\mu ^{2}\mathcal {A}^{*}\mathcal {A})^{-1}f\rangle _{L^{2}(\widehat{\pi })}. \end{aligned}$$

#### Remark 7

The proof of the preceding Proposition will show that $\mathcal {L}_{0}^{2}+\mu ^{2}\mathcal {A}^{*}\mathcal {A}$ is invertible on $L^2_0(\widehat{\pi })$ and that $(\mathcal {L}_{0}^{2}+\mu ^{2}\mathcal {A}^{*}\mathcal {A})^{-1}f \in \mathcal {D}(\mathcal {L}_0)$ for all $f \in L^2_0(\widehat{\pi })$.

To prove Proposition 2 we will make use of the *generator with reversed perturbation*

$$\begin{aligned} \mathcal {L}_{-}=\mathcal {L}_{0}-\mu \mathcal {A} \end{aligned}$$

and the *momentum flip operator*

51 $$\begin{aligned} P:L_{0}^{2}(\widehat{\pi }) \rightarrow L_{0}^{2}(\widehat{\pi }),\quad \phi (q,p) \mapsto \phi (q,-p). \end{aligned}$$

Clearly, $P^{2}=I$ and $P^{*}=P$. Further properties of $\mathcal {L}_{0}$, $\mathcal {A}$ and the auxiliary operators $\mathcal {L}_{-}$ and *P* are gathered in the following lemma:

#### Lemma 5

For all $\phi , \psi \in C^{\infty }(\mathbb {R}^{2d})\cap L^2(\widehat{\pi })$ the following holds:

- The generator $\mathcal {L}_{0}$ is symmetric in $L^2(\widehat{\pi })$ with respect to *P*:
  $$\begin{aligned} \langle \phi , P\mathcal {L}_{0}P \psi \rangle _{L^2(\widehat{\pi })}=\langle \mathcal {L}_{0} \phi , \psi \rangle _{L^2(\widehat{\pi })}. \end{aligned}$$
- The perturbation $\mathcal {A}$ is skewadjoint in $L^{2}(\widehat{\pi })$:
  $$\begin{aligned} \mathcal {A}^{*} = -\mathcal {A}. \end{aligned}$$
- The operators $\mathcal {L}_{0}$ and $\mathcal {A}$ commute:
  $$\begin{aligned}{}[\mathcal {L}_{0},\mathcal {A}]\phi =0. \end{aligned}$$
- The perturbation $\mathcal {A}$ satisfies
  $$\begin{aligned} P\mathcal {A}P\phi =\mathcal {A}\phi . \end{aligned}$$
- $$\mathcal {L}$$ and $$\mathcal {L}_{-}$$ commute,
  $$\begin{aligned}{}[\mathcal {L},\mathcal {L}_{-}]\phi = 0, \end{aligned}$$
  and the following relation holds:
  52 $$\begin{aligned} \langle \phi ,P\mathcal {L}P\psi \rangle _{L^{2}(\widehat{\pi })}=\langle \mathcal {L}_{-}\phi ,\psi \rangle _{ L^{2}(\widehat{\pi })}. \end{aligned}$$
- The operators $\mathcal {L}$, $\mathcal {L}_0$, $\mathcal {L}_{-}$, $\mathcal {A}$ and *P* leave the Hermite spaces $H_m$ invariant.

#### Remark 8

The claim (c) in the above lemma is crucial for our approach, which itself rests heavily on the fact that the $q-$ and $p-$perturbations match ($J_{1}=J_{2}$).

#### Proof of Lemma 5

The statement (a) is well-known and its proof can be found in [35, Sect. 2.2.3.1] for instance. The claim (b) follows by noting that the flow vector field $b(q,p)=(-Jq,-Jp)$ associated to $\mathcal {A}$ is divergence-free with respect to $\widehat{\pi }$, i.e. $\nabla \cdot (\widehat{\pi }b)=0$. Therefore, $\mathcal {A}$ is the generator of a strongly continuous unitary semigroup on $L^2(\widehat{\pi })$ and hence skewadjoint by Stone’s Theorem. The claims (c), (d) and (e) follow by direct computations which can be found in [41]. To prove (f) first notice that $\mathcal {L}$, $\mathcal {L}_0$ and $\mathcal {L}_{-}$ are of the form (36) and therefore leave the spaces $H_m$ invariant by Theorem 4. It follows immediately that also $\mathcal {A}$ leaves those spaces invariant. The fact that *P* leaves the spaces $H_m$ invariant follows directly by inspection of (49) and (51). $\square$

Now we proceed with the proof of Proposition 2:

#### Proof of Proposition 2

Since the potential *V* is quadratic, Assumption 1 clearly holds and thus Lemma 2 ensures that $\mathcal {L}$ and $\mathcal {L}_{-}$ are invertible on $L^2_{0}(\widehat{\pi })$ with

53 $$\begin{aligned} (-\mathcal {L})^{-1}=\int _0^\infty {e^{t\mathcal {L}}}\mathrm {d}t, \end{aligned}$$

and analogously for $\mathcal {L}_{-}^{-1}$. In particular, the asymptotic variance can be written as $\sigma _{f}^{2}=\langle f,(-\mathcal {L})^{-1}f\rangle _{L^{2}(\widehat{\pi })}.$ Due to the respresentation (53) and Theorem 4, the inverses of $\mathcal {L}$ and $\mathcal {L}_{-}$ leave the Hermite spaces $H_m$ invariant. We will prove the claim from Proposition 2 under the assumption that $Pf=f$ which includes the case $f=f(q)$. For the following calculations we will assume $f\in H_m$ for fixed $m \ge 1$. Combining statement (f) with (a) and (e) of Lemma 5 (and noting that $H_m \subset C^\infty (\mathbb {R}^{2d})\cap L^2(\widehat{\pi })$) we see that

54 $$\begin{aligned} P\mathcal {L}P=\mathcal {L}_{-}^{*} \quad \text{ and } \quad P\mathcal {L}_{0}P=\mathcal {L}_{0}^{*} \end{aligned}$$

when restricted to $H_m$. Therefore, the following calculations are justified:

$$\begin{aligned} \langle f,(-\mathcal {L})^{-1}f\rangle _{L^{2}(\widehat{\pi })}&= \frac{1}{2}\langle f,(-\mathcal {L})^{-1}f\rangle _{L^{2}(\widehat{\pi })}+\langle f,(-\mathcal {L}^{*})^{-1}f\rangle _{L^{2}(\widehat{\pi })} \\&=\frac{1}{2}\langle f,(-\mathcal {L})^{-1}f\rangle _{L^{2}(\widehat{\pi })}+\langle Pf,(-\mathcal {L}^{*})^{-1}Pf\rangle _{L^{2}(\widehat{\pi })}\\&=\frac{1}{2}\langle f,(-\mathcal {L})^{-1}f\rangle _{L^{2}(\widehat{\pi })}+\langle f,(-\mathcal {L}_{-})^{-1}f\rangle _{L^{2}(\widehat{\pi })}\\&=\frac{1}{2}\langle f,\left( (-\mathcal {L})^{-1}+(-\mathcal {L}_{-})^{-1}\right) f\rangle _{L^{2}(\widehat{\pi })}, \end{aligned}$$

where in the second line we have used the assumption $Pf=f$ and in the third line the properties $P^{2}=I$, $P^{*}=P$ and Eq. (54). Since $\mathcal {L}$ and $\mathcal {L}_{-}$ commute on $H_m$ according to Lemma 5(e),(f) we can write

$$\begin{aligned} (-\mathcal {L})^{-1}+(-\mathcal {L}_{-})^{-1} =\mathcal {L}_{-}(-\mathcal {L}\mathcal {L}_{-})^{-1}+\mathcal {L}(-\mathcal {L}\mathcal {L}_{-})^{-1} =-2\mathcal {L}_{0}(\mathcal {L}\mathcal {L}_{-})^{-1} \end{aligned}$$

for the restrictions on $H_m$, using $\mathcal {L}+\mathcal {L}_{-}=2\mathcal {L}_{0}$. We also have $\mathcal {L}\mathcal {L}_{-} =(\mathcal {L}_{0}+\mu \mathcal {A})(\mathcal {L}_{0}-\mu \mathcal {A}) =\mathcal {L}_{0}^{2}+\mu ^{2}\mathcal {A}^{*}\mathcal {A},$ since $\mathcal {L}_{0}$ and $\mathcal {A}$ commute. We thus arrive at the formula

55 $$\begin{aligned} \sigma _{f}^{2}=\langle f,-\mathcal {L}_{0}(\mathcal {L}_{0}^{2}+\mu ^{2}\mathcal {A}^{*}\mathcal {A})^{-1}f\rangle _{L^{2}(\widehat{\pi })}, \quad f\in H_m. \end{aligned}$$

Now since $(\mathcal {L}_{0}^{2}+\mu ^{2}\mathcal {A}^{*}\mathcal {A})^{-1}f = (\mathcal {L}\mathcal {L}_{-})^{-1}f \in \mathcal {D}(\mathcal {L}_{0})$ for all $f\in L^2(\widehat{\pi })$, it follows that the operator $-\mathcal {L}_{0}(\mathcal {L}_{0}^{2}+\mu ^{2}\mathcal {A}^{*}\mathcal {A})^{-1}$ is bounded. We can therefore extend formula (55) to the whole of $L^2(\widehat{\pi })$ by continuity, using the fact that $L^2_0(\widehat{\pi })=\bigoplus _{m\ge 1}H_m$. $\square$

Applying Proposition 2 we can analyse the behaviour of $\sigma _{f}^{2}$ in the limit of large perturbation strength $\mu \rightarrow \infty$. To this end, we introduce the orthogonal decomposition

56 $$\begin{aligned} L_{0}^{2}(\pi )=\ker (Jq\cdot \nabla _q) \oplus \ker (Jq\cdot \nabla _q)^{\perp }, \end{aligned}$$

where $Jq\cdot \nabla _q$ is understood as an unbounded operator acting on $L_0^2(\pi )$, obtained as the smallest closed extension of $Jq\cdot \nabla _q$ acting on $C^{\infty }_c(\mathbb {R}^d)$. In particular, $\ker (Jq\cdot \nabla _q)$ is a closed linear subspace of $L^2_0(\pi )$. Let $\varPi$ denote the $L_{0}^{2}(\pi )$-orthogonal projection onto $\ker (Jq\cdot \nabla _q)$. We will write $\sigma _{f}^{2}(\mu )$ to stress the dependence of the asymptotic variance on the perturbation strength. The following result shows that for large perturbations, the limiting asymptotic variance is always smaller than the asymptotic variance in the unperturbed case. Furthermore, the limit is given as the asymptotic variance of the projected observable $\varPi f$ for the unperturbed dynamics.

#### Theorem 5

Let $f\in L_{0}^{2}(\pi )$ (so in particular $f = f(q)$). Then $\lim _{\mu \rightarrow \infty }\sigma _{f}^{2}(\mu )=\sigma _{\varPi f}^{2}(0)\le \sigma _{f}^{2}(0).$

#### Remark 9

Note that the fact that the limit exists and is finite is nontrivial. In particular, as Fig. 1b, c demonstrate, it is often the case that $\lim _{\mu \rightarrow \infty }\sigma _{f}^{2}(\mu )=\infty$ if the condition $\mu =\nu$ is not satisfied.

#### Remark 10

The decomposition (56) can be interpreted in terms of the spectrum $\sigma (\mathcal {L})$ as follows: First observe that for functions *f* that only depend on *q*, $f \in \ker (Jq\cdot \nabla _q)$ is equivalent to $f \in \ker \mathcal {A}$. Let us denote by $\bar{\sigma } = \bigcap _{\mu \in \mathbb {R}} \sigma (\mathcal {L}_0 + \mu \mathcal {A})$ the part of $\sigma (\mathcal {L}_0)$ that is not affected by the perturbation and by

$$\begin{aligned} \bar{E} = \overline{{{\mathrm{span}}}\{f \in L^2(\hat{\pi }): \,\text {there exists } \lambda \in \bar{\sigma } \,\, \text {such that } \mathcal {L}_0 f = \lambda f \}} \subset L^2(\hat{\pi }) \end{aligned}$$

the corresponding subspace. Then it is straightforward to see that $\ker (\mathcal {A}) = \bar{E}$.^3^ In Fig. 2a, $\bar{\sigma }$ has been highlighted by diamonds.

#### Proof of Theorem 5

Note that $\mathcal {L}_{0}$ and $\mathcal {A}^{*}\mathcal {A}$ leave the Hermite spaces $H_m$ invariant and their restrictions to those spaces commute (see Lemma 5, (b), (c) and (f)). Furthermore, as the Hermite spaces $H_m$ are finite-dimensional, those operators have discrete spectrum. As $\mathcal {A}^{*}\mathcal {A}$ is nonnegative self-adjoint, there exists an orthogonal decomposition $L_{0}^{2}(\pi )=\bigoplus _{i}W_{i}$ into eigenspaces of the operator $-\mathcal {L}_{0}(\mathcal {L}_{0}^{2}+\mu ^{2}\mathcal {A}^{*}\mathcal {A})^{-1}$, the decomposition $\bigoplus W_i$ being finer then $\bigoplus H_m$ in the sense that every $W_i$ is a subspace of some $H_m$. Moreover, $-\mathcal {L}_{0}(\mathcal {L}_{0}^{2}+\mu ^{2}\mathcal {A}^{*}\mathcal {A})^{-1}\vert _{W_{i}}=-\mathcal {L}_{0}(\mathcal {L}_{0}^{2}+\mu ^{2}\lambda _{i})^{-1}\vert _{W_i},$ where $\lambda _{i}\ge 0$ is the eigenvalue of $\mathcal {A}^{*}\mathcal {A}$ associated to the subspace $W_{i}$. Consequently, formula (50) can be written as

57 $$\begin{aligned} \sigma _{f}^{2}=\sum _{i}\langle f_{i},-\mathcal {L}_{0}(\mathcal {L}_{0}^{2}+\mu ^{2}\lambda _{i})^{-1}f_{i}\rangle _{L^{2}(\widehat{\pi })}, \end{aligned}$$

where $f=\sum _{i}f_{i}$ and $f_{i}\in W_{i}$. Let us assume now without loss of generality that $W_{0}=\ker \mathcal {A}^{*}\mathcal {A}$, so in particular $\lambda _{0}=0$. Then clearly

$$\begin{aligned} \lim _{\mu \rightarrow \infty }\sigma _{f}^{2}=2\langle f_{0},-\mathcal {L}_{0}(\mathcal {L}_{0}^{2})^{-1}f_{0}\rangle _{L^{2}(\widehat{\pi })}=2\langle f_{0},(-\mathcal {L}_{0})^{-1}f_{0}\rangle _{L^{2}(\widehat{\pi })}=\sigma _{f_{0}}^{2}(0). \end{aligned}$$

Now notice that $W_{0}=\ker \mathcal {A}^{*}\mathcal {A}=\ker \mathcal {A}$, showing the equality in the claim. It remains to show that $\sigma _{\varPi f}^{2}(0)\le \sigma _{f}^{2}(0)$. To see this, we write

$$\begin{aligned} \sigma _{f}^{2}(0)&=2\left\langle f,(-\mathcal {L}_{0})^{-1}f\rangle _{L^{2}(\widehat{\pi })}=2\langle \varPi f+(1-\varPi )f,(-\mathcal {L}_{0})^{-1}\big (\varPi f+(1-\varPi )f\big )\right\rangle _{L^{2}(\widehat{\pi })}\\&=\sigma _{\varPi f}^{2}(0)+\sigma _{(1-\varPi )f}^{2}(0)+R, \end{aligned}$$

where

$$\begin{aligned} R=2\langle \varPi f,(-\mathcal {L}_{0})^{-1}(1-\varPi )f\rangle _{L^{2}(\widehat{\pi })}+2\langle (1-\varPi )f,(-\mathcal {L}_{0})^{-1}\varPi f\rangle _{L^{2}(\widehat{\pi })}. \end{aligned}$$

Note that since we only consider observables that do not depend on *p*, $\varPi f\in \ker (Jq\cdot \nabla _q)$ and $(1-\varPi )f\in \bigoplus _{i\ge 1}W_{i}$. Since $\mathcal {L}_{0}$ commutes with $\mathcal {A}$, it follows that $(-\mathcal {L}_{0})^{-1}$ leaves both $W_{0}$ and $\bigoplus _{i\ge 1}W_{i}$ invariant. Therefore, as the latter spaces are orthogonal to each other, it follows that $R=0$, from which the result follows. $\square$

From Theorem 5 it follows that in the limit as $\mu \rightarrow \infty$, the asymptotic variance $\sigma _f^2(\mu )$ is not decreased by the perturbation if $f \in \ker (Jq \cdot \nabla _q)$. In fact, this result also holds true non-asymptotically, i.e. observables in $\ker (Jq \cdot \nabla _q)$ are not affected at all by the perturbation:

#### Lemma 6

Let $f\in \ker (Jq\cdot \nabla _q)$. Then $\sigma ^2_f(\mu ) = \sigma ^2_f(0)$ for all $\mu \in \mathbb {R}$.

#### Proof

From $f \in \ker (Jq\cdot \nabla _q)$ it follows immediately that $f \in \ker \mathcal {A}^{*}\mathcal {A}$. Then the claim follows from the expression (57). $\square$

#### Example 1

Recall the case of observables of the form $f(q)=q\cdot Kq+l\cdot q+C$ with $K\in \mathbb {R}_{sym}^{d\times d}$, $l\in \mathbb {R}^{d}$ and $C\in \mathbb {R}$ from Sect. 4.1. If $[J,K]=0$ and $l\in \ker J$, then $f\in \ker (Jq\cdot \nabla _q)$ as

$$\begin{aligned} Jq\cdot \nabla _{q}(q\cdot Kq+l\cdot q+C)=2Jq\cdot Kq+Jq\cdot l=q\cdot (KJ-JK)q-q\cdot Jl=0. \end{aligned}$$

From the preceding lemma it follows that $\sigma _{f}^{2}(\mu )=\sigma _{f}^{2}(0)$ for all $\mu \in \mathbb {R},$ showing that the assumption in Theorem 3 does not exclude nontrivial cases.

The following result shows that the dynamics (34) is particularly effective for antisymmetric observables (at least in the limit of large perturbations):

#### Proposition 3

Let $f\in L_{0}^{2}(\pi )$ satisfy $f(-q)=-f(q)$ and assume that $\ker J=\{0\}$. Furthermore, assume that the eigenvalues of *J* are rationally independent, i.e.

58 $$\begin{aligned} \sigma (J)=\{\pm i\lambda _{1},\pm i\lambda _{2},\ldots ,\pm i\lambda _d\} \end{aligned}$$

with $\lambda _{i}\in \mathbb {R}_{>0}$ and $\sum _i k_i \lambda _i \ne 0$ for all $(k_1,\ldots ,k_d)\in \mathbb {Z}^d\setminus (0,\ldots ,0)$. Then $\lim _{\mu \rightarrow \infty }\sigma _{f}^{2}(\mu )=0$.

#### Proof of Proposition 3

The claim would immediately follow from $f\in \ker (Jq\cdot \nabla )^{\perp }$ according to Theorem 5, but that does not seem to be so easy to prove directly. Instead, we again make use of the Hermite polynomials.

Recall from the proof of Proposition 2 that ${{\mathrm{\mathcal {L}}}}$ is invertible on $L_{0}^{2}(\widehat{\pi })$ and its inverse leaves the Hermite spaces $H_m$ invariant. Consequently, the asymptotic variance of an observable $f\in L_{0}^{2}(\widehat{\pi })$ can be written as

59 $$\begin{aligned} \sigma _{f}^{2} = \langle f,(-\mathcal {L})^{-1}f\rangle _{L^{2}(\widehat{\pi })} = \sum _{m=1}^{\infty }\langle \varPi _{m}f,(-\mathcal {L}\vert _{H_{m}})^{-1}\varPi _{m}f\rangle _{L^2(\widehat{\pi })}, \end{aligned}$$

where $\varPi _{m}:L_{0}^{2}(\widehat{\pi })\rightarrow H_{m}$ denotes the orthogonal projection onto $H_{m}$. From (49) it is clear that $g_{a}$ is symmetric for $\vert \alpha \vert$ even and antisymmetric for $\vert \alpha \vert$ odd. Therefore, from *f* being antisymmetric it follows that $f\in \bigoplus _{m\ge 1,m\,\text {odd}}H_{m}.$ In view of (45), ((c)) and (58) the spectrum of $\mathcal {L}_{\vert H_{m}}$ can be written as

60 $$\begin{aligned} \sigma (\mathcal {L}\vert _{H_{m}})= & {} \left\{ \mu \sum _{j=1}^{2d}\alpha _{j}\beta _{j}+C_{\alpha ,\gamma }:\,\vert \alpha \vert =m,\,\beta _{j}\in \sigma (J)\right\} \nonumber \\= & {} \left\{ i\mu \sum _{j=1}^{d}(\alpha _{j}-\alpha _{j+d})\lambda _{j}+C_{\alpha ,\gamma }:\,\vert \alpha \vert =m\right\} \end{aligned}$$

with appropriate real constants $C_{\alpha ,\gamma }\in \mathbb {R}$ that depend on $\alpha$ and $\gamma$, but not on $\mu$. For $\vert \alpha \vert =\sum _{j=1}^{2d} \alpha _j=m$ odd, we have that

61 $$\begin{aligned} \sum _{j=1}^{d}(\alpha _{j}-\alpha _{j+d})\lambda _{j} \ne 0. \end{aligned}$$

Indeed, assume to the contrary that the above expression is zero. Then it would follow that $\alpha _j = \alpha _{j+d}$ for all $j=1,\ldots ,d$ by rational independence of $\lambda _1,\ldots ,\lambda _d$ and $\vert m \vert$ would have to be even. From (60) and (61) it is clear that

$$\begin{aligned} \sup \left\{ r>0 : B(0,r) \cap \sigma (\mathcal {L}\vert _{H_m}) = \emptyset \right\} \xrightarrow {\mu \rightarrow \infty } \infty , \end{aligned}$$

where *B*(0, *r*) denotes the ball of radius *r* centered at the origin in $\mathbb {C}$. Consequently, the spectral radius of $(-\mathcal {L}\vert _{H_m})^{-1}$ and hence $(-\mathcal {L}\vert _{H_m})^{-1}$ itself converges to zero as $\mu \rightarrow \infty$. The result then follows from (59). $\square$

#### Remark 11

The idea of the preceding proof can be explained using Fig. 2a and Remark 10. The eigenvalues in the fixed spectrum $\bar{E}$ (on the real axis, highlighted by diamonds) correspond to Hermite polynomials of even order. The independence condition on the eigenvalues of *J* prevents cancellations that would lead to fixed eigenvalues associated to Hermite polynomials of odd order. Therefore, antisymmetric observables are orthogonal to $\bar{E} = \ker \mathcal {A}$.

The following corollary gives a version of the converse of Proposition 3 and provides further intuition into the mechanics of the variance reduction achieved by the perturbation.

#### Corollary 1

Let $f\in L_{0}^{2}(\pi )$ and assume that $lim_{\mu \rightarrow \infty }\sigma _{f}^{2}(\mu )=0$. Then

$$\begin{aligned} \int _{B(0,r)}f\mathrm {dq=0} \end{aligned}$$

for all $r\in (0,\infty )$, where *B*(0, *r*) denotes the ball centered at 0 with radius *r*.

#### Proof

According to Theorem 5, $\lim _{\mu \rightarrow \infty }\sigma _{f}^{2}(\mu )=0$ implies $\sigma _{\varPi f}^{2}(0)=0$. We can write

62 $$\begin{aligned} \sigma _{\varPi f}^{2}(0) = \langle \varPi f, (-\mathcal {L}_0)^{-1}\varPi f \rangle _{ L^{2}(\widehat{\pi })} = \frac{1}{2}\langle \varPi f, \left( (-\mathcal {L}_0)^{-1}+(-\mathcal {L}^{*}_0)^{-1}\right) \varPi f \rangle _{ L^{2}(\widehat{\pi })} \end{aligned}$$

and recall from the proof of Proposition 2 that $(-\mathcal {L}_0)^{-1}$ and $(-\mathcal {L}^{*}_0)^{-1}$ leave the Hermite spaces $H_m$ invariant. Therefore $\ker \left( (-\mathcal {L}_0)^{-1}+(-\mathcal {L}^{*}_0)^{-1}\right) = {0}$ in $L^2_0(\widehat{\pi })$, and in particular $\sigma _{\varPi f}^{2}(0)=0$ implies $\varPi f = 0$, which in turn shows that $f\in \ker (Jq\cdot \nabla )^{\perp }$. From $(Jq\cdot \nabla )^{*} = -Jq\cdot \nabla$, it follows that

63 $$\begin{aligned} \ker (Jq\cdot \nabla )^{\perp }=\overline{{{\mathrm{im}}}(Jq\cdot \nabla )^{*}}=\overline{{{\mathrm{im}}}(Jq\cdot \nabla )}. \end{aligned}$$

Hence, there exists a sequence $(\phi _n)_n\in C_c^{\infty }(\mathbb {R}^d)$ such that $Jq\cdot \nabla \phi _n \rightarrow f$ in $L^2(\pi )$. Taking a subsequence if necessary, we can assume that the convergence is pointwise $\pi$-almost everywhere and that the sequence is pointwise bounded by a function in $L^1(\pi )$. Since *J* is antisymmetric, we have that $Jq\cdot \nabla \phi _n=\nabla \cdot (\phi _n Jq)$. Now Gauss’s theorem yields

$$\begin{aligned} \int _{B(0,r)}f\mathrm {d}q=\int _{B(0,r)}\nabla \cdot (\phi Jq)\mathrm {d}q=\int _{\partial B(0,r)}\phi Jq\cdot \mathrm {d}n, \end{aligned}$$

where *n* denotes the outward normal to the sphere $\partial B(0,r)$. This quantity is zero due to the orthogonality of *Jq* and *n*, and so the result follows from Lebesgue’s dominated convergence theorem. $\square$

### Optimal Choices of *J* for Quadratic Observables

Assume $f\in L_{0}^{2}(\pi )$ is given by $f(q)=q\cdot Kq+l\cdot q -{{\mathrm{Tr}}}K$, with $K\in \mathbb {R}_{sym}^{d\times d}$ and $l\in \mathbb {R}^{d}$ (note that the constant term is chosen such that $\pi (f)=0$). Our objective is to choose *J* in such a way that $\lim _{\mu \rightarrow \infty }\sigma _{f}^{2}(\mu )$ becomes as small as possible. To stress the dependence on the choice of *J*, we introduce the notation $\sigma _{f}^{2}(\mu ,J)$. Also, we denote the orthogonal projection onto $(\ker J)^{\perp }$ by $\varPi ^{\perp }_{\ker J}$.

#### Lemma 7

(Zero variance limit for linear observables). Assume $K=0$ and $\varPi ^{\perp }_{\ker J}l=0$. Then

$$\begin{aligned} \lim _{\mu \rightarrow \infty }\sigma _{f}^{2}(\mu ,J)=0. \end{aligned}$$

#### Proof

According to Theorem 5, we have to show that $\varPi f=0$, where $\varPi$ is the $L^{2}(\pi )$-orthogonal projection onto $\ker (Jq\cdot \nabla )$. Let us thus use (63) and prove that $f\in \overline{{{\mathrm{im}}}(Jq\cdot \nabla )}.$ Indeed, since $\varPi ^{\perp }_{\ker J}{l}=0$, by Fredholm’s alternative there exists $u \in \mathbb {R}^d$ such that $Ju=l$. Now define $\phi \in L_{0}^{2}(\pi )$ by $\phi (q)=-u\cdot q,$ leading to $f=Jq\cdot \nabla \phi ,$ so the result follows. $\square$

#### Lemma 8

(Zero variance limit for purely quadratic observables.) Let $l=0$ and consider the decomposition $K=K_{0}+K_{1}$ into the traceless part $K_{0}=K-\frac{{{\mathrm{Tr}}}K}{d}\cdot I$ and the trace-part $K_{1}=\frac{{{\mathrm{Tr}}}K}{d}\cdot I.$ For the corresponding decomposition of the observable

$$\begin{aligned} f(q)=f_{0}(q)+f_{1}(q)=q\cdot K_{0}q+q\cdot K_{1}q-{{\mathrm{Tr}}}K \end{aligned}$$

the following holds:

- There exists an antisymmetric matrix *J* such that $\lim _{\mu \rightarrow \infty }\sigma _{f_{0}}^{2}(\mu ,J)=0,$ and there is an algorithmic way (see Algorithm 1) to compute an appropriate *J* in terms of *K*.
- The trace-part is not effected by the perturbation, i.e. $\sigma _{f_{1}}^{2}(\mu ,J)=\sigma _{f_{1}}^{2}(0)$ for all $\mu \in \mathbb {R}$.

#### Proof

To prove the first claim, according to Theorem 5 it is sufficient to show that $f_{0}\in \ker (Jq\cdot \nabla )^{\perp }=\overline{{{\mathrm{im}}}(Jq\cdot \nabla )}$. Let us consider the function $\phi (q)=q\cdot Aq$, with $A\in \mathbb {R}_{sym}^{d\times d}$. It holds that $Jq\cdot \nabla \phi ={2}q\cdot (J^{T}Aq)=q\cdot [A,J]q.$ The task of finding an antisymmetric matrix *J* such that $\lim _{\mu \rightarrow \infty }\sigma _{f_{0}}^{2}(\mu ,J)=0$ can therefore be accomplished by constructing an antisymmetric matrix *J* such that there exists a symmetric matrix *A* with the property $K_{0}=[A,J]$. Given any traceless matrix $K_{0}$ there exists an orthogonal matrix $U\in O(\mathbb {R}^{d})$ such that $UK_{0}U^{T}$ has zero entries on the diagonal, and that *U* can be obtained in an algorithmic manner (see for example [29] or [22, Chap. 2, Sect. 2, Problem 3]) Assume thus that such a matrix $U\in O(\mathbb {R}^{d})$ has been found and choose real numbers $a_1,\ldots ,a_d \in \mathbb {R}$ such that $a_{i}\ne a_{j}$ if $i\ne j$. We now set $\bar{A}={{\mathrm{diag}}}(a_{1},\ldots ,a_{n}),$ and

64 $$\begin{aligned} \bar{J}_{ij}= {\left\{ \begin{array}{ll} \frac{(UK_{0}U^{T})_{ij}}{a_{i}-a_{j}} &{} \text {if } i\ne j, \\ 0 &{} \text {if } i=j. \\ \end{array}\right. } \end{aligned}$$

Observe that since $UK_{0}U^{T}$ is symmetric, $\bar{J}$ is antisymmetric. A short calculation shows that $[\bar{A},\bar{J}]= UK_{0}U^{T}$. We can thus define $A=U^{T}\bar{A}U$ and $J=U^{T}\bar{J}U$ to obtain $[A,J]=K_0$. Therefore, the *J* constructed in this way indeed satisfies (4.4). For the second claim, note that $f_{1}\in \ker (Jq\cdot \nabla )$, since $Jq\cdot \nabla \left( q\cdot \frac{{{\mathrm{Tr}}}K}{d}q\right) =2\frac{{{\mathrm{Tr}}}K}{d}q\cdot Jq=0$ due to the antisymmetry of *J*. The result then follows from Lemma 6. $\square$

We would like to stress that the perturbation *J* constructed in the previous lemma is far from unique due to the freedom of choice of *U* and $a_1,\ldots ,a_d \in \mathbb {R}$ in its proof. However, it is asymptotically optimal:

#### Corollary 2

In the setting of Lemma 8 the following holds:

$$\begin{aligned} \min _{J^T=-J}\left( \lim _{\mu \rightarrow \infty } \sigma ^2_{f}(\mu ,J)\right) =\sigma ^2_{f_1}(0). \end{aligned}$$

#### Proof

The claim follows immediately since $f_{1}\in \ker (Jq\cdot \nabla )$ for arbitrary antisymmetric *J* as shown in (4.4), and therefore the contribution of the trace part $f_1$ to the asymptotic variance cannot be reduced by any choice of *J* according to Lemma 6. $\square$

As the proof of Lemma 8 is constructive, we obtain the following algorithm for determining optimal perturbations for quadratic observables:

#### Algorithm 1

Given $K\in \mathbb {R}_{sym}^{d\times d}$, determine an optimal antisymmetric perturbation *J* as follows:

1. Set $$K_{0}=K-\frac{{{\mathrm{Tr}}}K}{d}\cdot I.$$
2. Find $U\in O(\mathbb {R}^{d})$ such that $UK_{0}U^{T}$ has zero entries on the diagonal.
3. Choose $a_{i}\in \mathbb {R},$ $i=1,\ldots d$ such that $a_{i}\ne a_{j}$ for $i\ne j$ and set
  $$\begin{aligned} \bar{J}_{ij}=\frac{(UK_{0}U^{T})_{ij}}{a_{i}-a_{j}} \end{aligned}$$
  for $i\ne j$ and $\bar{J}_{ii}=0$ otherwise.
4. Set $$J=U^{T}\bar{J}U$$.

#### Remark 12

In [14], the authors consider the task of finding optimal perturbations *J* for the nonreversible overdamped Langevin dynamics given in (15). In the Gaussian case this optimization problem turns out be equivalent to the one considered in this section. Indeed, equation (39) of [14] can be rephrased as $f \in \ker (Jq\cdot \nabla )^{\perp }.$ Therefore, Algorithm 1 and its generalization Algorithm 2 (described in Sect. 4.5) can be used without modifications to find optimal perturbations of overdamped Langevin dynamics.

### Gaussians with Arbitrary Covariance and Preconditioning

In this section we extend the results of the preceding sections to the case when the target measure $\pi$ is given by a Gaussian with arbitrary covariance, i.e. $V(q)=\frac{1}{2}q\cdot Sq$ with $S\in \mathbb {R}_{sym}^{d\times d}$ symmetric and positive definite. The dynamics (8) then takes the form

65 $$\begin{aligned} \mathrm {d}q_{t} =M^{-1}p_{t}\mathrm {d}t-\mu J_{1}Sq_{t}\mathrm {d}t, \quad \! \mathrm {d}p_{t} =-Sq_{t}\mathrm {d}t-\nu J_{2}M^{-1}p_{t}\mathrm {d}t-\varGamma M^{-1}p_{t}\mathrm {d}t+\sqrt{2\varGamma }\mathrm {d}W_{t}. \end{aligned}$$

The key observation is now that the choices $M=S$ and $\varGamma =\gamma S$ together with the transformation $\widetilde{q}=S^{1/2}q$ and $\widetilde{p}=S^{-1/2}p$ lead to the dynamics

66 $$\begin{aligned} \mathrm {d}\widetilde{q}_{t}&=\widetilde{p}_{t}\mathrm {d}t-\mu S^{1/2}J_{1}S^{1/2}\widetilde{q}_{t}\mathrm {d}t,\nonumber \\ \mathrm {d}\widetilde{p}_{t}&=-\widetilde{q}_{t}\mathrm {d}t-\mu S^{-1/2}J_{2}S^{-1/2}\widetilde{p}_{t}\mathrm {d}t-\gamma \widetilde{p}_{t}\mathrm {d}t+\sqrt{2\gamma }\mathrm {d}W_{t}, \end{aligned}$$

which is of the form (34) if $J_{1}$ and $J_{2}$ obey the condition $SJ_{1}S=J_{2}$. Clearly the dynamics (66) is ergodic with respect to a Gaussian measure with unit covariance, in the following denoted by $\widetilde{\pi }$. The connection between the asymptotic variances associated to (65) and (66) is as follows:

For an observable $f\in L_{0}^{2}(\pi )$ we can write

$$\begin{aligned} \sqrt{T}\bigg (\frac{1}{T}\int _{0}^{T}f(q_{s})\mathrm {d}s-\pi (f)\bigg )=\sqrt{T}\bigg (\frac{1}{T}\int _{0}^{T}\widetilde{f}(\widetilde{q}_{s})\mathrm {d}s-\widetilde{\pi }(\widetilde{f})\bigg ), \end{aligned}$$

where $\widetilde{f}(q)=f(S^{-1/2}q)$. Therefore, the asymptotic variances satisfy $\sigma _{f}^{2}=\widetilde{\sigma }_{\widetilde{f}}^{2},$ where $\widetilde{\sigma }_{\widetilde{f}}^{2}$ denotes the asymptotic variance of the process $(\widetilde{q}_{t})_{t\ge 0}$. Because of this, the results from the previous sections generalise to (65), subject to the condition that the choices $M=S$, $\varGamma =\gamma S$ and $SJ_{1}S=J_{2}$ are made. We formulate our results in this general setting as corollaries:

#### Corollary 3

Consider the dynamics

67 $$\begin{aligned} \mathrm {d}q_{t}= & {} M^{-1}p_{t}\mathrm {d}t-\mu J_{1}\nabla V(q_{t})\mathrm {d}t,\nonumber \\ \mathrm {d}p_{t}= & {} -\nabla V(q_{t})\mathrm {d}t-\mu J_{2}M^{-1}p_{t}\mathrm {d}t-\varGamma M^{-1}p_{t}\mathrm {d}t+\sqrt{2\varGamma }\mathrm {d}W_{t}, \end{aligned}$$

with $V(q)=\frac{1}{2}q\cdot Sq$. Assume that $M=S$, $\varGamma =\gamma S$ with $\gamma > \sqrt{2}$ and $SJ_{1}S=J_{2}$. Let $f\in L^{2}(\pi )$ be an observable of the form

68 $$\begin{aligned} f(q)=q\cdot Kq+l\cdot q+C \end{aligned}$$

with $K\in \mathbb {R}_{sym}^{d\times d}$, $l\in \mathbb {R}^{d}$ and $C\in \mathbb {R}$. If at least one of the conditions $KJ_{1}S\ne SJ_{1}K$ and $l \notin \ker J$ is satisfied, then the asymptotic variance is at a local maximum for the unperturbed sampler, i.e.

$$\begin{aligned} \left. \partial _{\mu }\sigma _{f}^{2}\right| _{\mu =0}=0\qquad \text{ and } \qquad \left. \partial _{\mu }^{2}\sigma _{f}^{2}\right| _{\mu =0}<0. \end{aligned}$$

#### Proof

Note that

$$\begin{aligned} \widetilde{f}(q)=f(S^{-1/2}q)=q\cdot S^{-1/2}KS^{-1/2}q+S^{-1/2}l\cdot q+C=q\cdot \widetilde{K}q+\widetilde{l}\cdot q+C \end{aligned}$$

is again of the form (68) (where in the last equality, $\widetilde{K}=S^{-1/2}KS^{-1/2}$ and $\widetilde{l}=S^{-1/2}l$ have been defined). From (66), (4.5) and Theorem 3 the claim follows if at least one of the conditions $[\widetilde{K},S^{1/2}J_{1}S^{1/2}]\ne 0$ and $\widetilde{l}\notin \ker (S^{1/2}J_{1}S^{1/2})$ is satisfied. The first of those can easily seen to be equivalent to $S^{-1/2}(KJS-SJK)S^{-1/2}\ne 0,$ which is equivalent to $KJ_{1}S\ne SJ_{1}K$ since *S* is nondegenerate. The second condition is equivalent to $S^{1/2}J_{1}l\ne 0,$ which is equivalent to $J_{1}l\ne 0,$ again by nondegeneracy of *S*. $\square$

#### Corollary 4

Assume the setting from the previous corollary and denote by $\varPi$ the orthogonal projection onto $\ker (J_{1}Sq\cdot \nabla )$. For $f\in L^{2}(\pi )$ it holds that $\lim _{\mu \rightarrow \infty }\sigma _{f}^{2}(\mu )=\sigma _{\varPi f}^{2}(0)\le \sigma _{f}^{2}(0).$

#### Proof

Theorem 5 implies $\lim _{\mu \rightarrow \infty }\widetilde{\sigma }_{\widetilde{f}}^{2}(\mu )=\widetilde{\sigma }_{\widetilde{\varPi }\widetilde{f}}^{2}(0)\le \widetilde{\sigma }_{\widetilde{f}}^{2}(0)$ for the transformed system (66). Here $\widetilde{f}(q)=f(S^{-1/2}q)$ is the transformed observable and $\widetilde{\varPi }$ denotes $L^{2}(\pi )$-orthogonal projection onto $\ker (S^{1/2}J_{1}S^{1/2}q\cdot \nabla )$. According to (4.5), it is sufficient to show that $(\varPi f)\circ S^{-1/2}=\widetilde{\varPi }\widetilde{f}$. This however follows directly from the fact that the linear transformation $\phi \mapsto \phi \circ S^{1/2}$ maps $\ker (S^{1/2}J_{1}S^{1/2}q\cdot \nabla )$ bijectively onto $\ker (J_{1}Sq\cdot \nabla )$. $\square$

Let us also reformulate Algorithm 1 for the case of a Gaussian with arbitrary covariance.

#### Algorithm 2

Given $K,S\in \mathbb {R}_{sym}^{d\times d}$ with $f(q)=q\cdot Kq$ and $V(q)=\frac{1}{2}q\cdot Sq$ (assuming *S* is nondegenerate), determine optimal perturbations $J_{1}$ and $J_{2}$ as follows:

1. Set $$\widetilde{K}=S^{-1/2}KS^{-1/2}$$ and $$\widetilde{K}_{0}=\widetilde{K}-\frac{{{\mathrm{Tr}}}\widetilde{K}}{d}\cdot I$$.
2. Find $U\in O(\mathbb {R}^{d})$ such that $U\widetilde{K}_{0}U^{T}$ has zero entries on the diagonal.
3. Choose $a_{i}\in \mathbb {R}$, $i=1,\ldots ,d$ such that $a_{i}\ne a_{j}$ for $i\ne j$ and set
  $$\begin{aligned} \bar{J}_{ij}=\frac{(U\widetilde{K}_{0}U^{T})_{ij}}{a_{i}-a_{j}}. \end{aligned}$$
4. Set $$\widetilde{J}=U^{T}\bar{J}U$$.
5. Put $$J_{1}=S^{-1/2}\widetilde{J}S^{-1/2}$$ and $$J_{2}=S^{1/2}JS^{1/2}$$.

Finally, we obtain the following optimality result from Lemma 7 and Corollary 2.

#### Corollary 5

Let $f(q)=q\cdot Kq+l\cdot q-{{\mathrm{Tr}}}K$ and assume that $\varPi ^{\perp }_{\ker J}l=0$. Then

$$\begin{aligned} \min _{J_1^T=-J_1,\, J_2=SJ_1 S}\left( \lim _{\mu \rightarrow \infty } \sigma ^2_{f}(\mu ,J_1,J_2)\right) =\sigma ^2_{f_1}(0), \end{aligned}$$

where $f_{1}(q)=q\cdot K_{1}q$, $K_{1}=\frac{{{\mathrm{Tr}}}(S^{-1}K)}{d}S$. Optimal choices for $J_{1}$ and $J_{2}$ can be obtained using Algorithm 2.

#### Remark 13

Since in Sect. 4.1 we analysed the case where $J_{1}$ and $J_{2}$ are proportional, we are not able to drop the restriction $J_{2}=SJ_{1}S$ from the above optimality result. Analysis of completely arbitrary perturbations will be the subject of future work.

#### Remark 14

The choices $M=S$ and $\varGamma =\gamma S$ have been introduced to make the perturbations considered in this article lead to samplers that perform well in terms of reducing the asymptotic variance. However, adjusting the mass and friction matrices according to the target covariance in this way (i.e. $M=S$ and $\varGamma =\gamma S$) is a popular way of preconditioning the dynamics, see for instance [18] and, in particular mass-tensor molecular dynamics [6]. Here we will present an argument why such a preconditioning is indeed beneficial in terms of the convergence rate of the dynamics. Let us first assume that *S* is diagonal, i.e. $S={{\mathrm{diag}}}(s^{(1)},\ldots ,s^{(d)})$ and that $M={{\mathrm{diag}}}(m^{(d)},\ldots ,m^{(d)})$ and $\varGamma ={{\mathrm{diag}}}(\gamma ^{(d)},\ldots ,\gamma ^{(d)})$ are chosen diagonally as well. Then (65) decouples into one-dimensional SDEs of the following form:

69 $$\begin{aligned} \mathrm {d}q_{t}^{(i)} =\frac{1}{m^{(i)}}p_{t}^{(i)}\mathrm {d}t,\quad \mathrm {d}p_{t}^{(i)} =-s^{(i)}q_{t}^{(i)}\mathrm {d}t-\frac{\gamma ^{(i)}}{m^{(i)}}p_{t}^{(i)}\mathrm {d}t+\sqrt{2\gamma ^{(i)}}\mathrm {d}W_{t},\quad i=1,\ldots ,d. \end{aligned}$$

Let us write those Ornstein–Uhlenbeck processes as

70 $$\begin{aligned} \mathrm {d}X_{t}^{(i)}=-B^{(i)}X_{t}^{(i)}\mathrm {d}t+\sqrt{2Q^{(i)}}\mathrm {d}W_{t}^{(i)}, \quad B^{(i)}=\left( \begin{array}{c@{\quad }c} 0 &{} -\frac{1}{m^{(i)}}\\ s^{(i)} &{} \frac{\gamma ^{(i)}}{m^{(i)}} \end{array}\right) ,\quad Q^{(i)}=\left( \begin{array}{c@{\quad }c} 0 &{} 0\\ 0 &{} \gamma ^{(i)} \end{array}\right) . \end{aligned}$$

As in Sect. 4.2, the rate of the exponential decay of (70) is equal to $\min \text {Re}\,\sigma (B^{(i)})$. A short calculation shows that the eigenvalues of $B^{(i)}$ are given by

$$\begin{aligned} \lambda _{1,2}^{(i)}=\frac{\gamma ^{(i)}}{2m^{(i)}}\pm \sqrt{\bigg (\frac{\gamma ^{(i)}}{2m^{(i)}}\bigg )^{2}-\frac{s^{(i)}}{m^{(i)}}}. \end{aligned}$$

Therefore, the rate of exponential decay is maximal when

71 $$\begin{aligned} \bigg (\frac{\gamma ^{(i)}}{2m^{(i)}}\bigg )^{2}-\frac{s^{(i)}}{m^{(i)}}=0, \end{aligned}$$

in which case it is given by

$$\begin{aligned} (\lambda ^{(i)})^{*}=\sqrt{\frac{s^{(i)}}{m^{(i)}}}. \end{aligned}$$

Naturally, it is reasonable to choose $m^{(i)}$ in such a way that the exponential rate $(\lambda ^{(i)})^{*}$ is the same for all *i*, leading to the restriction $M=cS$ with $c>0$. Choosing *c* small will result in fast convergence to equilibrium, but also make the dynamics (69) quite stiff, requiring a very small timestep $\varDelta t$ in a discretisation scheme. The choice of *c* will therefore need to strike a balance between those two competing effects. The constraint (71) then implies $\varGamma =2cS$. By a coordinate transformation, the preceding argument also applies if *S*, *M* and $\varGamma$ are diagonal in the same basis, and of course *M* and $\varGamma$ can always be chosen that way. Numerical experiments show that it is possible to increase the rate of convergence to equilibrium even further by choosing *M* and $\varGamma$ nondiagonally with respect to *S* (although only by a small margin). A clearer understanding of this is a topic of further investigation.

## Numerical Experiments: Diffusion Bridge Sampling

### Numerical Scheme

In this section we introduce a splitting scheme for simulating the perturbed underdamped Langevin dynamics given by Eq. (8). In the unpertubed case, i.e. when $J_{1}=J_{2}=0$, the *BAOAB* scheme (see [33] and references therein) has proven to be efficient for computing long time ergodic averages with respect to *q*-dependent observables. Motivated by this, we introduce the following perturbed scheme, introducing additional Runge-Kutta integration steps:

72a $$\begin{aligned} p_{n+1/2}= & {} p_{n}-\frac{1}{2}\varDelta t\nabla V(q_{n}), \end{aligned}$$

72b $$\begin{aligned} q_{n+1/2}= & {} q_{n}+\frac{1}{2}\varDelta t\cdot M^{-1}p_{n+1/2},\end{aligned}$$

72c $$\begin{aligned} q'_{n+1/2}= & {} RK_{4}\left( \frac{1}{2}\varDelta t,q_{n+1/2}\right) ,\end{aligned}$$

72d $$\begin{aligned} \hat{p}= & {} \exp (-\varDelta t(\varGamma M^{-1}+\nu J_{2}M^{-1}))p_{n+1/2}+\sqrt{I-e^{-2\varGamma \varDelta t}}\mathcal {N}(0,1),\end{aligned}$$

72e $$\begin{aligned} q''_{n+1/2}= & {} RK_{4}\left( \frac{1}{2}\varDelta t,q'_{n+1/2}\right) ,\end{aligned}$$

72f $$\begin{aligned} q_{n+1}= & {} q''_{n+1/2}+\frac{1}{2}\varDelta t\cdot M^{-1}\hat{p},\end{aligned}$$

72g $$\begin{aligned} p_{n+1}= & {} \hat{p}-\frac{1}{2}\varDelta t\cdot \nabla V(q_{n+1}), \end{aligned}$$

where $RK_{4}(\varDelta t,q_{0})$ refers to fourth order Runge-Kutta integration of the ODE

73 $$\begin{aligned} \dot{q}=-J_{1}\nabla V(q), \quad q(0)=q_0 \end{aligned}$$

up until time $\varDelta t$. We remark that the $J_{2}$-perturbation is linear and can therefore be included in the *O*-part without much computational overhead. We emphasize the fact that many different splitting schemes could be investigated: although the BAOAB-scheme works well for unperturbed Langevin dynamics, it is not clear whether this remains true for the perturbed dynamics. Moreover, the perturbations introduced by $J_1$ and $J_2$ can be added in various places. Other discretisation schemes for the ODE (73) could be useful as well, for instance one could use a symplectic integrator, using the Hamiltonian structure of (73). However, since *V* as the Hamiltonian for (73) is not separable in general, such a symplectic integrator would have to be implicit. Note that (72c) and (72e) could be merged since (72e) commutes with (72d). In this paper, we content ourselves with the above scheme for our numerical experiments. Investigation of optimal numerical schemes for perturbed Langevin dynamics is an interesting problem for further research.

#### Remark 15

The aformentioned schemes lead to an error in the approximation for $\pi (f)$, since the invariant measure $\pi$ is not preserved exactly by the numerical scheme. In practice, the *BAOAB*-scheme can therefore be accompanied by an accept-reject Metropolis step as in [40], leading to an unbiased estimate of $\pi (f)$, albeit with an inflated variance. In this case, after every rejection the momentum variable has to be flipped ($p\mapsto -p$) in order to keep the correct invariant measure. We note here that our perturbed scheme can be ’Metropolized’ in a similar way by ’flipping’ the matrices $J_{1}$ and $J_{2}$ after every rejection ($J_{1}\mapsto -J_{1}$ and $J_{2}\mapsto -J_{2})$ and using an appropriate (volume-preserving and time-reversible) integrator for the dynamics given by (73). Implementations of this idea are the subject of ongoing work. See [47] for a similar approach to nonreversible overdamped Langevin dynamics.

### Diffusion Bridge Sampling

To numerically test our analytical results, we will apply the dynamics (8) to sample a measure on path space associated to a diffusion bridge. Specifically, consider the SDE

$$\begin{aligned} \mathrm {d}X_{s}=-\nabla U(X_{s})\mathrm {d}s+\sqrt{2\beta ^{-1}}\mathrm {d}W_{s}, \end{aligned}$$

with $X_{s}\in \mathbb {R}^{n}$, $\beta >0$ and the potential $U:\mathbb {R}^{n}\rightarrow \mathbb {R}$ obeying adequate growth and smoothness conditions (see [24], Sect. 5 for precise statements). The law of the solution to this SDE conditioned on the events $X(0)=x_{-}$ and $X(s_{+})=x_{+}$ is a probability measure $\pi$ on $L^{2}([0,s_{+}],\mathbb {R}^{n})$ which poses a challenging and important sampling problem, especially if *U* is multimodal. This setting has been used as a test case for sampling probability measures in high dimensions (see for example [9] and [45]). For a more detailed introduction (including applications) see [11] and for a rigorous theoretical treatment the papers [11, 24–26].

In the case $U\equiv 0$, it can be shown that the law of the conditioned process is given by a Gaussian measure $\pi _{0}$ with mean zero and precision operator $\mathcal {S}=-\frac{\beta }{2}\varDelta$ on the Sobolev space $H^{1}([0,s_{+}],\mathbb {R}^{d})$ equipped with appropriate boundary conditions. The general case can then be understood as a perturbation thereof: The measure $\pi$ is absolutely continuous with respect to $\pi _{0}$ with Radon-Nikodym derivative

74 $$\begin{aligned} \frac{\mathrm {d}\pi }{\mathrm {d}\pi _{0}}\propto \exp \big (-\varPsi \big ), \end{aligned}$$

where

$$\begin{aligned} \varPsi (x)=\frac{\beta }{2}\int _{0}^{s_{+}}G(x(s),\beta )\mathrm {d}s \quad \text{ and }\quad G(x,\beta )=\frac{1}{2}\vert \nabla U(x)\vert ^{2}-\frac{1}{\beta }\varDelta U(x). \end{aligned}$$

We will make the choice $x_{-}=x_{+}=0$, which is possible without loss of generality as explained in [10, Remark 3.1], leading to Dirichlet boundary conditions on $[0,s_+]$ for the precision operator $\mathcal {S}$. Furthermore, we choose $s_{+}=1$ and discretise the ensuing *s*-interval [0, 1] according to

$$\begin{aligned}{}[0,1]=[0,s_{1})\cup [s_{1},s_{2})\cup \ldots \cup [s_{n-1},s_{n})\cup [s_{n},1] \end{aligned}$$

in an equidistant way with stespize $s_{j+1}-s_{j}\equiv \delta =\frac{1}{d+1}$. Functions on this grid are determined by the values $x(s_1)=x_1,\ldots ,x(s_n)=x_n$, recalling that $x(0)=x(1)=0$ by the Dirichlet boundary conditions. We discretise the functional $\varPsi$ as

$$\begin{aligned} \tilde{\varPsi }(x_{1},\ldots ,x_{n}) =\frac{\beta }{2}\delta \sum _{i=1}^{d}G(x_{i},\beta ) =\frac{\beta }{2}\delta \sum _{i=1}^{d}\Big ({\frac{1}{2}}(U'(x_{i})^{2}-\frac{1}{\beta }U''(x_{i})\Big ), \end{aligned}$$

such that its gradient is given by

$$\begin{aligned} (\nabla \tilde{\varPsi })_{i}=\frac{\beta }{2}\delta \big (2U'(x_{i})U''(x_{i})-\frac{1}{\beta }U'''(x_{i})\big ),\quad i=1,\ldots ,d. \end{aligned}$$

We denote by $A_{\delta }$ the discretised Dirichlet Laplacian on [0, 1] with stepsize $\delta$. Following (74), the discretised target measure $\widehat{\pi }$ has the form

$$\begin{aligned} \widehat{\pi }=\frac{1}{Z}e^{-V}\mathrm {d}x \quad \text{ with } \quad V(x)=\tilde{\varPsi }(x)-\frac{\beta \delta }{4}x\cdot A_{\delta }x,\quad x\in \mathbb {R}^{d}. \end{aligned}$$

In the following we will consider the case $n=1$ with potential $U:\mathbb {R}\rightarrow \mathbb {R}$ given by $U(x)=\frac{1}{2}(x^{2}-1)^{2}$ and set $\beta =1$. To test our algorithm we adjust the parameters *M*, $\varGamma$, $J_{1}$ and $J_{2}$ according to the recommended choice in the Gaussian case, (27), where we take $S=\frac{\beta }{2}\delta \cdot A_{\delta }$ as the precision operator of the Gaussian target. We will consider the linear observable $f_{1}(x)=l\cdot x$ with $l=(1,\ldots ,1)$ and the quadratic observable $f_{2}(x)=\vert x\vert ^{2}$. In a first experiment we adjust the perturbations $J_{1}$ and $J_{2}$ to the observable $f_{2}$ according to Algorithm 2. The dynamics (8) is integrated using the splitting scheme introduced in Sect. 5.1 with a stepsize of $\varDelta t=10^{-4}$ over the time interval [0, *T*] with $T=10^{2}$. Furthermore, we choose initial conditions $q_0=(1,\ldots ,1)$, $p_0=(0,\ldots ,0)$ and introduce a burn-in time $T_{0}=1$, i.e. we take the estimator to be $\hat{\pi }(f)\approx \frac{1}{T-T_{0}}\int _{T_{0}}^{T}f(q_{t})\mathrm {d}t.$ We compute the variance of the above estimator from $N=500$ realisations and compare the results for different choices of the friction coefficient $\gamma$ and of the perturbation strength $\mu$.

**Fig. 4:**
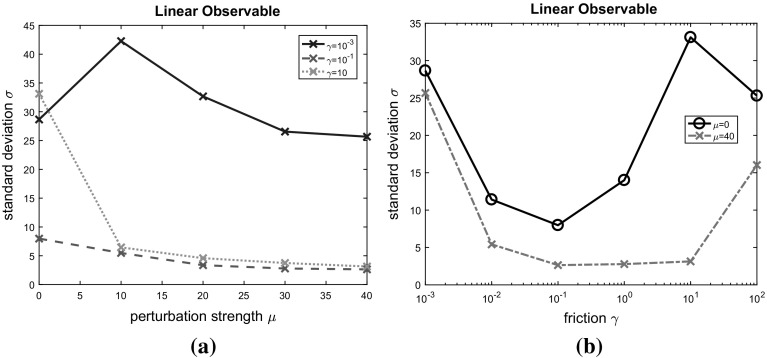
Standard deviation of $\hat{\pi }(f)$ for a linear observable as a function of friction $\gamma$ and perturbation strength $\mu$

**Fig. 5:**
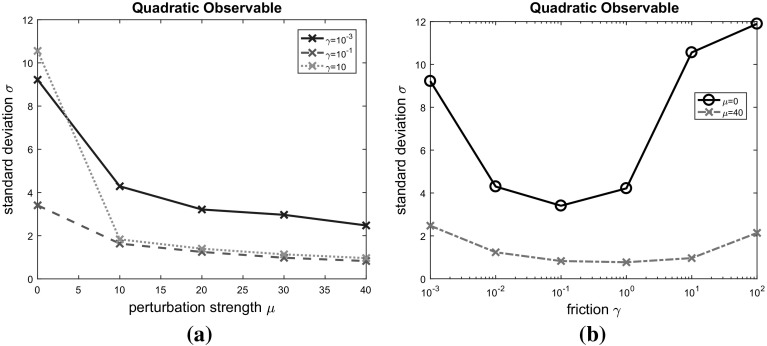
Standard deviation of $\hat{\pi }(f)$ for a quadratic observable as a function of friction $\gamma$ and perturbation strength $\mu$

The numerical experiments show that the perturbed dynamics generally outperform the unperturbed dynamics independently of the choice of $\mu$ and $\gamma$, both for linear and quadratic observables. One notable exception is the behaviour of the linear observable for small friction $\gamma = 10^{-3}$ (see Fig. 4a), where the asymptotic variance initially increases for small perturbation strengths $\mu$. This does not contradict our analytical results, since the small perturbation results Theorem 3 and Corollary 3 are only valid if $\gamma > \sqrt{2}$. We remark here that the condition $\gamma >\sqrt{2}$, while necessary for the theoretical results from Sect. 4.1, is not a very advisable choice in practice (at least in this experiment), since Figs. 4b and 5b clearly indicate that the optimal friction is around $\gamma \approx 10^{-1}$. Interestingly, the problem of choosing a suitable value for the friction coefficient coefficient $\gamma$ becomes mitigated by the introduction of the perturbation: While the performance of the unperturbed sampler depends quite sensitively on $\gamma$, the asymptotic variance of the perturbed dynamics is a lot more stable with respect to variations of $\gamma$. A somewhat surprising phenomenon is that the standard deviation $\sigma$ associated to the linear observable decays in the range $\gamma \in [10,100]$ for the unperturbed sampler (see Fig. 4b). We confirmed this behaviour by further numerical experiments and remark that as the target measure $\hat{\pi }$ is fairly complicated, convexity of the function $\gamma \mapsto \sigma$ should not be expected.

In the regime of growing values of $\mu$, the experiments confirm the results from Sect. 4.3, i.e. the asymptotic variance approaches a limit that is smaller than the asymptotic variance of the unperturbed dynamics.

As a final remark we report our finding that the performance of the sampler for the linear observable is qualitatively independent of the choice of $J_1$ [as long as $J_2$ is adjusted according to (27)]. This result is in alignment with Propostion 3 which predicts good properties of the sampler for antisymmetric observables. In contrast to this, a judicious choice of $J_1$ is critical for quadratic observables. In particular, applying Algorithm 2 significantly improves the performance of the perturbed sampler in comparison to choosing $J_1$ arbitrarily.

## Outlook and Future Work

A new family of Langevin samplers was introduced in this paper. These new SDE samplers consist of perturbations of the underdamped Langevin dynamics (that is known to be ergodic with respect to the canonical measure), where auxiliary drift terms in the equations for both the position and the momentum are added, in a way that the perturbed family of dynamics is ergodic with respect to the same (canonical) distribution. These new Langevin samplers were studied in detail for Gaussian target distributions where it was shown, using tools from spectral theory for differential operators, that an appropriate choice of the perturbations in the equations for the position and momentum can improve the performance of the Langvin sampler, at least in terms of reducing the asymptotic variance. The performance of the perturbed Langevin sampler to non-Gaussian target densities was tested numerically on the problem of diffusion bridge sampling.

The work presented in this paper can be improved and extended in several directions. First, a rigorous analysis of the new family of Langevin samplers for non-Gaussian target densities is needed. The analytical tools developed in [14] can be used as a starting point. Furthermore, the study of the actual computational cost and its minimization by an appropriate choice of the numerical scheme and of the perturbations in position and momentum would be of interest to practitioners. In addition, the analysis of our proposed samplers can be facilitated by using tools from symplectic and differential geometry. Finally, combining the new Langevin samplers with existing variance reduction techniques such as zero variance MCMC, preconditioning/Riemannian manifold MCMC can lead to sampling schemes that can be of interest to practitioners, in particular in molecular dynamics simulations. All these topics are currently under investigation.

## Footnotes

1 In fact, using the results from [8], we could consider observables in $L^2(\pi )$. However, we will not extend this point further in this paper.

2 Notice that $\sqrt{\left( \frac{\gamma }{2}\right) ^2-1}$ is understood to be a complex number for $\gamma < 2$.

3 Indeed, the fact that $f\in \ker \mathcal {A}$ is equivalent to $f \in \bar{E}$ is easy to check if *f* is an eigenvector of $\mathcal {L}_0$ (recall that *f* is then an eigenvector of $\mathcal {L}_0 +\mu \mathcal {A}$ as well, using Lemma 5(c) The claim then follows by extending linearly.
